# Supplementary material for: A spatiotemporal model of firearm ownership in the United States
Source: Patterns (N Y). 2022 Jun 29;3(8):100546. doi: 10.1016/j.patter.2022.100546 (PMC9403408; doi:10.1016/j.patter.2022.100546)
Supplement: Document S1. Figures S1–S6, Tables S1–S6, and supplemental experimental procedures [file mmc1.pdf]

Patterns, Volume *3*

Supplemental information

A spatiotemporal model

of firearm ownership in the United States

Roni Barak-Ventura, Manuel Ruiz Marín, and Maurizio Porfiri

Supplemental procedures

S1. State-level time series

We present the raw time series for background checks (BC; Fig. S1), background checks per capita (BCC; Fig. S2), and fraction of suicides that were committed with firearms (SF; Fig. S3), in each state. We also include the prediction of our model for the fraction of firearm owners in each state, overlaid with the fraction of firearm owners reports by the Gallup Poll Social Series (GPSS) every October (FO; Fig. S4).

S2. Exploring different spatial weight matrices

Next, we summarize the results for calibration of our model with different spatial weight matrices (Table S1). Specifically, we considered spatial weight matrices that encapsulate the relationships between states based on their distance (D), area (A), population size (p), gross domestic product (GDP), and presence of a border (B). This endeavor was carried out with the underlying notion that states that are more proximate, vast, populated, economically productive, or share a border, may exert more influence on other states.
To facilitate the selection of a weight matrix for our analysis, we examined the autoregressive terms and residuals of each model. Inspection of the results revealed that the terms τ and η were not significantly different from zero for any of the models. The autoregressive term ρ emerged only for the models that contain D or B. Among those models, the lowest σ^2 was registered for the one with W containing $\frac{p_j}{D_{i,j}}$ in its elements. Thus, this spatial weight matrix was selected for our study.

S3. Evaluating the model output

Once the model output was obtained, the predicted FO in each state every October was compared to the corresponding fraction of firearm owners reported by the GPSS by computing the sum of squared errors (SSE) and the mean of squared errors (MSE). The smallest errors occurred in highly populated states such as New York, California, and Florida. In contrast, the largest errors appeared in the least populated states, such as South Dakota, Vermont, and Wyoming (Table S9).

S4. Pre-processing time series

State-level BC and BCC exhibited non-stationarity and strong seasonality in all states. Therefore, before computing transfer entropy between any pair of time series, it was imperative to remove those patterns in order to prevent erroneous inference of causality. We determined stationarity of raw time series for each variable under consideration (BC, BCC, SF, and FO) in each state using an Augmented Dickey-Fuller test (Table S9). The test revealed that both variables were majorly non-stationary: only 11 states exhibited stationarity with respect to BC, 12 showed stationarity with respect to BCC, and 14 with respect to fraction of SF. In order to treat all states equally, all time series were seasonally adjusted and detrended. The Augmented Dickey-Fuller test was applied following this procedure to confirm that the time series were now stationary at a significance level of 0.05.
The time series of mass shootings was adapted from data collected by Mother Jones.[1] The data consist of 64 mass shooting events from January 2000 to December 2017 (Table S4). The monthly time series of mass shootings in our analysis was binary (consisting of zeros and ones), where 0 denoted no mass shootings took place and 1 reflected at least one mass shooting.[2]

S5. Testing for time series Markovianity

Before proceeding to transfer entropy analysis, we confirmed that the time series in our analysis are Markovian. Within the framework of mutual information, we computed

$$I(X_t; X_{t-2}|X_{t-1}, X_{t-3}) = H(X_t|X_{t-1}, X_{t-3}) - H(X_t|X_{t-1}, X_{t-2}, X_{t-3}) \quad \text{(S1)}$$

for each time series. Should the time series be Markovian, the quantity in equation S1 would be equal to zero. To verify that the value was not significantly different from zero, we performed a permutation test where we shuffled the time series of X_{t-2} and computed conditional mutual information as in equation S1. We repeated this procedure 50,000 times to obtain 50,000 values of mutual information. This surrogate distribution represented the conditional mutual information that would result from chance. We tested whether the observed value was not in the right tail of the surrogate distribution, within its 95th percentile. For the time series of BC, BCC, SF, and FO, we measured mutual information of magnitudes 0.0230, 0.0207, 0.0036, and 0.0147 bits, respectively. Those measurements were not significantly different from zero ($p = 0.1629$, $p = 0.2085$, $p = 0.9044$, and $p = 0.3832$, respectively). For the time series of MS and MO, mutual information assumed values of 0.0193 and 0.0249, respectively. These values were statistically indistinguishable from zero ($p = 0.2875$ and $p = 0.1351$, respectively).

S6. Testing for contemporaneous effects

In addition to assessing memory within time series, we also confirmed that the selection of one month's time step between measurements of our time series is appropriate. If causal relationships were to exist on a time interval smaller than a month, then spurious links could be inferred through conditional transfer entropy.[3] To exclude the possibility of such interactions, we tested for contemporaneous effects in each pair of variables X and Y within a triad. Specifically, we computed mutual information between contemporaneous time series X and Y at time step t, conditioned on the their past and the past of Z

$$I(X_t; Y_t|X_{t-1}, Y_{t-1}, Z_{t-1}) = H(X_t|X_{t-1}, Y_{t-1}, Z_{t-1}) - H(X_t|Y_t, X_{t-1}, Y_{t-1}, Z_{t-1}). \quad \text{(S2)}$$

In the absence of contemporaneous effects, the quantity in equation S2 should be equal to zero. To test whether this quantity was not statistically different from zero, we performed a permutation test. We shuffled the entire time series of X and Y such that any potential association between them is disrupted, and computed conditional mutual information as in equation S2. We computed 50,000 values of mutual information and generated a surrogate distribution representing the conditional mutual information from chance. Values that exceeded the 95th percentile of the surrogate distribution would be deemed significantly different from zero.

Within the benchmark triad of BC, MS, and MO, 0.0176 bits were computed between BC and MS, 0.0307 between BC and MO, and 0.0136 between MS and MO. None of these values was significantly different from zero ($p = 0.8306$, $p = 0.4376$, and $p = 0.9156$, respectively). For the triad containing BCC instead of BC, mutual information amounted to 0.0105 bits between BCC and MS, 0.0185 between BCC and MO, and 0.0094 between MS and MO. Here too, mutual information values were not significantly different from zero ($p = 0.9607$, $p = 0.7858$, and $p = 0.9724$, respectively). In the triad involving SF, MS, and MO, mutual information was measured at 0.0373 bits, 0.0359 bits, and 0.0297 bits for the links between SF and MS, SF and MO, and MS and MO, respectively. None of these measurements was statistically different from zero ($p = 0.3030$, $p = 0.3152$, and $p = 0.4890$, respectively). Finally, for the triad containing our measurement of FO, mutual information was 0.0288 bits between FO and MS, 0.0030 between FO and MO, and 0.0061 between MS and MO. None of those values was significantly different from zero ($p = 0.5142$, $p = 0.9900$, and $p = 0.9900$, respectively).

S7. Conditional transfer entropy with time delays

To challenge our model's output, we revisited one of our previous studies where causal links between firearm prevalence, MS, and MO were quantified and investigated.[2] Specifically, we computed transfer entropy between pairs of national-level time series and found four causal links: MO$\rightarrow$ BC|MS, FO$\rightarrow$ MS|MO, FO$\rightarrow$ MO|MS, and MO$\rightarrow$ FO|MS. In addition, we explored the possibility of delayed interactions between the variables by computing conditional transfer entropy with delays,

$$\mathrm{TE}_{Y\rightarrow X|Z} = I(X_{t+1}; Y_{t-\delta_Y}|X_t, Z_{t-\delta_Z}) = H(X_{t+1}|X_t, Z_{t-\delta_Z}) - H(X_{t+1}|X_t, Y_{t-\delta_Y}, Z_{t-\delta_Z}). \quad \text{(S3)}$$

where δ_Y and δ_Z are the delays used for Y and Z, respectively. In this setting, one delay can be used for the source variable (Y) and a different delay can be considered for the variable conditioned upon (Z). For each pairwise interaction, we independently varied δ_Y and δ_Z from 0 to 11 and computed the corresponding delayed conditional transfer entropy to obtain 12$\times$12 values. For example, considering transfer entropy from mass shootings (Y) to background checks (X) conditioned on media output (Z), we computed 12$\times$12 values of transfer entropy for delays in mass shootings and media output.

In Fig. S5, we highlight the results of these analyses for the links we found to be causal (MO$\rightarrow$ BC|MS, FO$\rightarrow$ MS|MO, FO$\rightarrow$ MO|MS, and MO$\rightarrow$ FO|MS). In each plots with blue markers on the left, we show the mean of 12 transfer entropy values with fixed delays on the source variable ranging from 0 to 11. The vertical bars extending from the markers represent the standard deviation when the delay on the conditional variable varies from 0 to 11. Similarly, in each of the plots with red markers, we show the means of 12 transfer entropy values with fixed delays on the conditional variable. There, vertical bars represent the standard deviation when delays are applied on the source variable.

For MO$\rightarrow$ BC|MS, FO$\rightarrow$ MS|MO, and MO$\rightarrow$ FO|MS, the largest transfer entropy value was observed for a delay of 0. For FO$\rightarrow$ MO|MS, transfer entropy is maximized for delays of 0 and 2 months. We further investigated the nature of the 2-months delay by examining transfer entropy on a state-level. Specifically, we hypothesized that the media's association with firearm prevalence would differ between permissive and restrictive states. In permissive states, there is more room for firearm legislation and MO on firearm control would focus on legislation in those states, whereas firearm control would be only marginally discussed with a longer delay in restrictive states. Thus, following our cluster analysis in,[4] we identified California, Connecticut, Illinois, Maryland, Massachusetts, New Jersey, and New York as restrictive, and all other states as permissive. For each delay ranging from 0 to 11, we computed a mean value of transfer entropy for all restrictive states (Fig. S6a) and for all permissive states (Fig. S6b), weighted by each state's population size. We found that transfer entropy from FO to MO is maximized for permissive states without a delay.

S8. Conditional transfer entropy in the absence of τ and η

In our prediction of FO, we accounted for all calibrated coefficients to improve the model's prediction. However, one might suggest that including coefficients that are not significantly different from zero in the model would create an over-specified model. To confirm that the inclusion of such terms does not compromise our inference of causal relationship, we generated a time series of firearm ownership on a national level between January 2000 and December 2017 with our model, specifying $\tau = 0$ and $\eta = 0$. We used the model output in transfer entropy analysis within the triad of FO, MS, and MO (Table S5) and verified that the results are similar to the findings in the main manuscript (Table 2).

S9. Conditional transfer entropy in the absence of spatial interactions

Finally, we aimed to demonstrate that spatial regression in our model gave rise to causal links in transfer entropy analysis. To this end, we inferred firearm ownership in each state between January 2000 and October 2019, using the calibrated parameters of a null model with $W = 0$ (first row in Table S1). In the absence of spatial interactions between states, the time series we generated linearly combined the

background checks per capita and fraction of suicides with firearms within a state to compute its own firearm ownership. We used the model output in transfer entropy analysis within the triad of FO/MS/MO and found that none of the links is significantly different from chance (Table S6). This result indicates that spatial interactions are crucial for the detection of causal links.

References

1. Follman, M., Aronsen, G., Pan, D. (2021), US Mass Shootings, 1982–2021: Data From Mother Jones' Investigation. URL `https://www.motherjones.com/politics/2012/12/mass-shootings-mother-jones-full-data/`.
2. Porfiri, M., Sattanapalle, R.R., Nakayama, S., Macinko, J., Sipahi, R. (2019). Media coverage and firearm acquisition in the aftermath of a mass shooting. Nature Human Behaviour, 3, 913–921, URL `http://dx.doi.org/10.1038/s41562-019-0636-0`.
3. Runge, J. (2020). Discovering contemporaneous and lagged causal relations in autocorrelated nonlinear time series datasets. In Conference on Uncertainty in Artificial Intelligence (PMLR), pp. 1388–1397.
4. Porfiri, M., Barak-Ventura, R., Marín, M.R. (2020). Self-protection versus fear of stricter firearm regulations: examining the drivers of firearm acquisitions in the aftermath of a mass shooting. Patterns, 1, 100082, URL `http://dx.doi.org/10.1016/j.patter.2020.100082`.

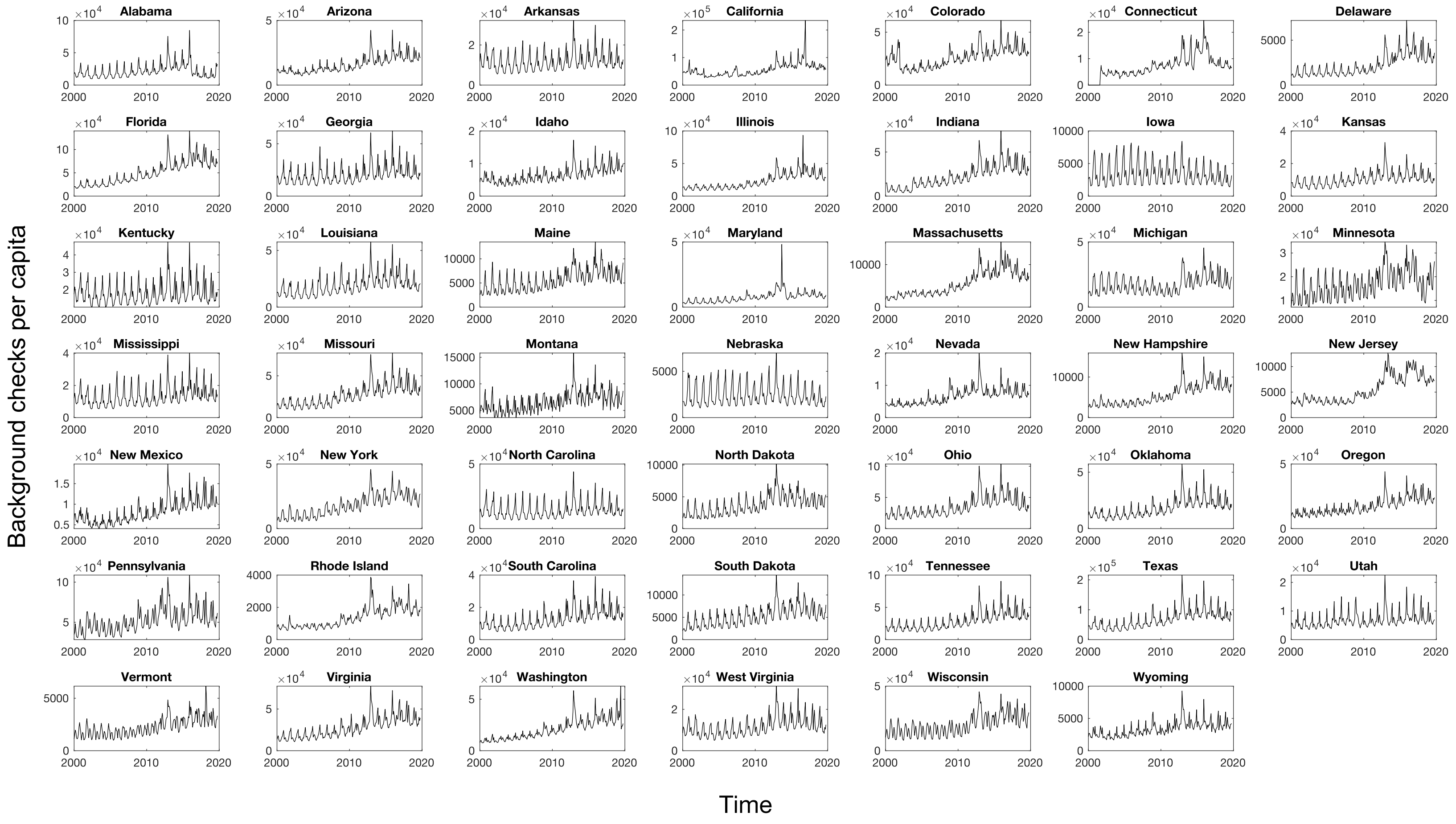

Figure S1: State-level background checks. Each panel displays the time series for monthly background checks in each U.S. state considered in the analysis. Alaska and Hawaii were excluded from the analysis due to missing data.

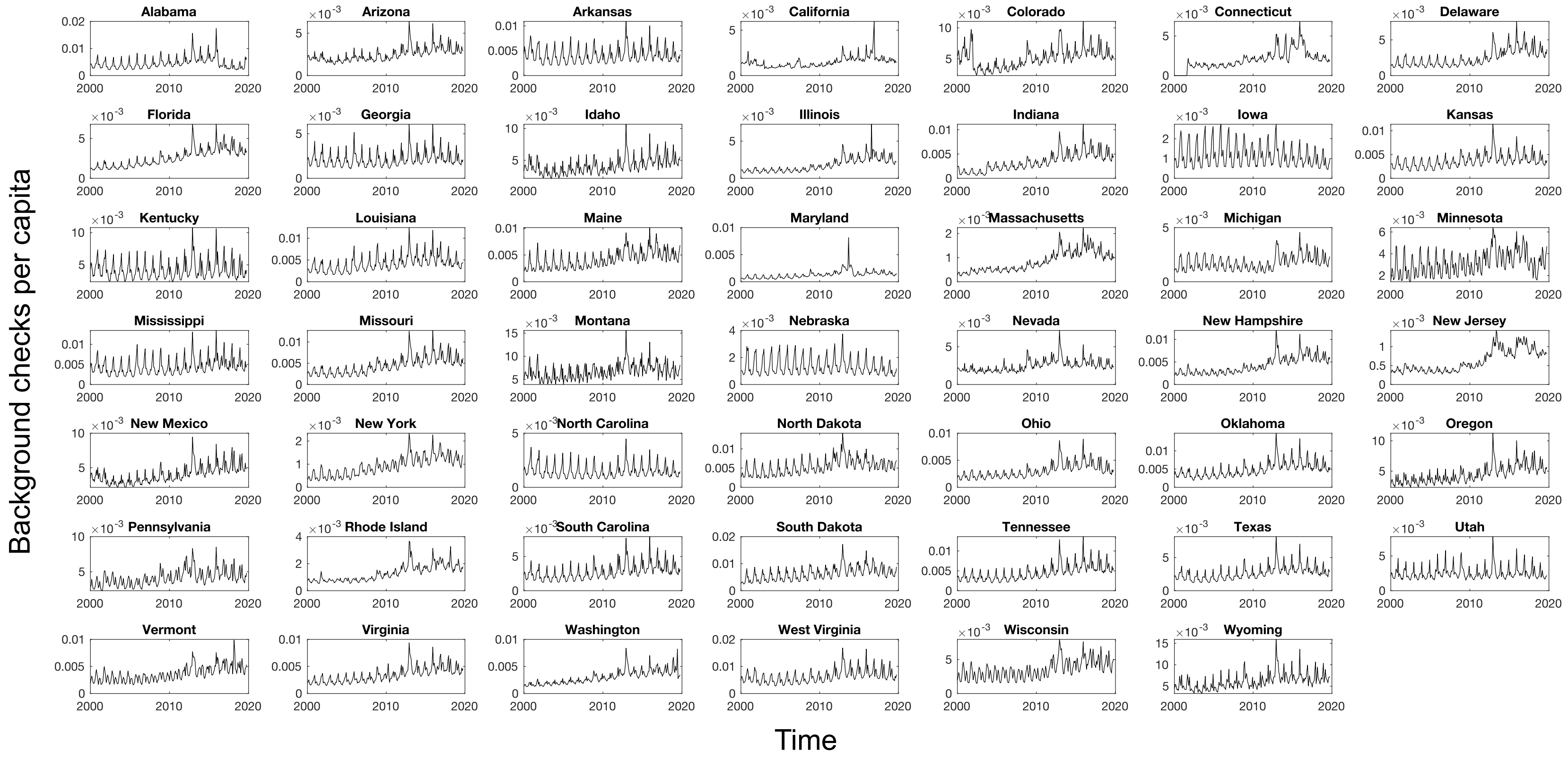

Figure S2: State-level background checks per capita. Each panel displays the time series for monthly background checks per capita in each U.S. state considered in the analysis. Alaska and Hawaii were excluded from the analysis due to missing data.

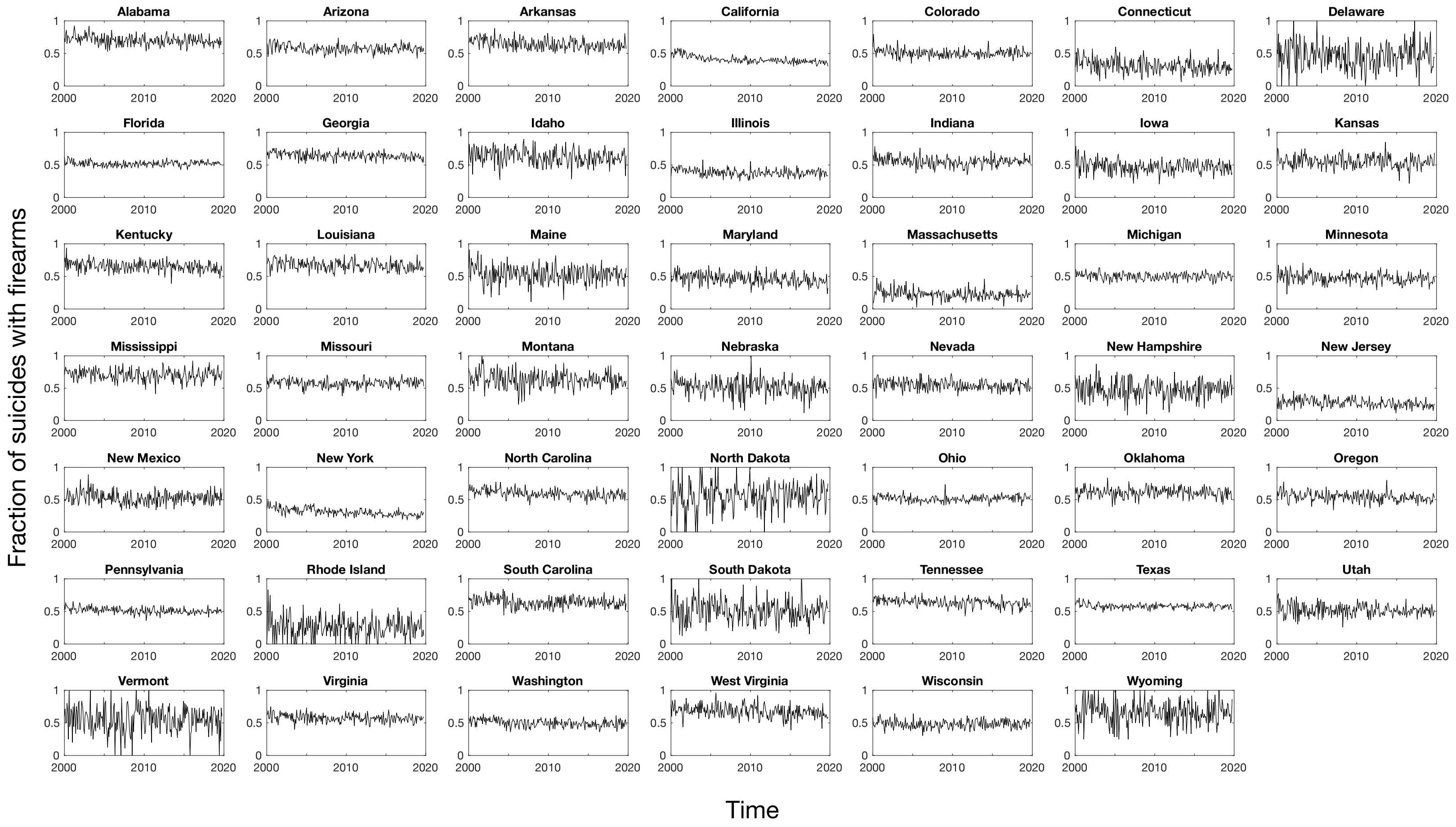

Figure S3: State-level suicides with firearms. Each panel displays the time series for the monthly fraction of suicides committed with firearms in each U.S. state considered in the analysis. Alaska and Hawaii were excluded from the analysis due to missing data.

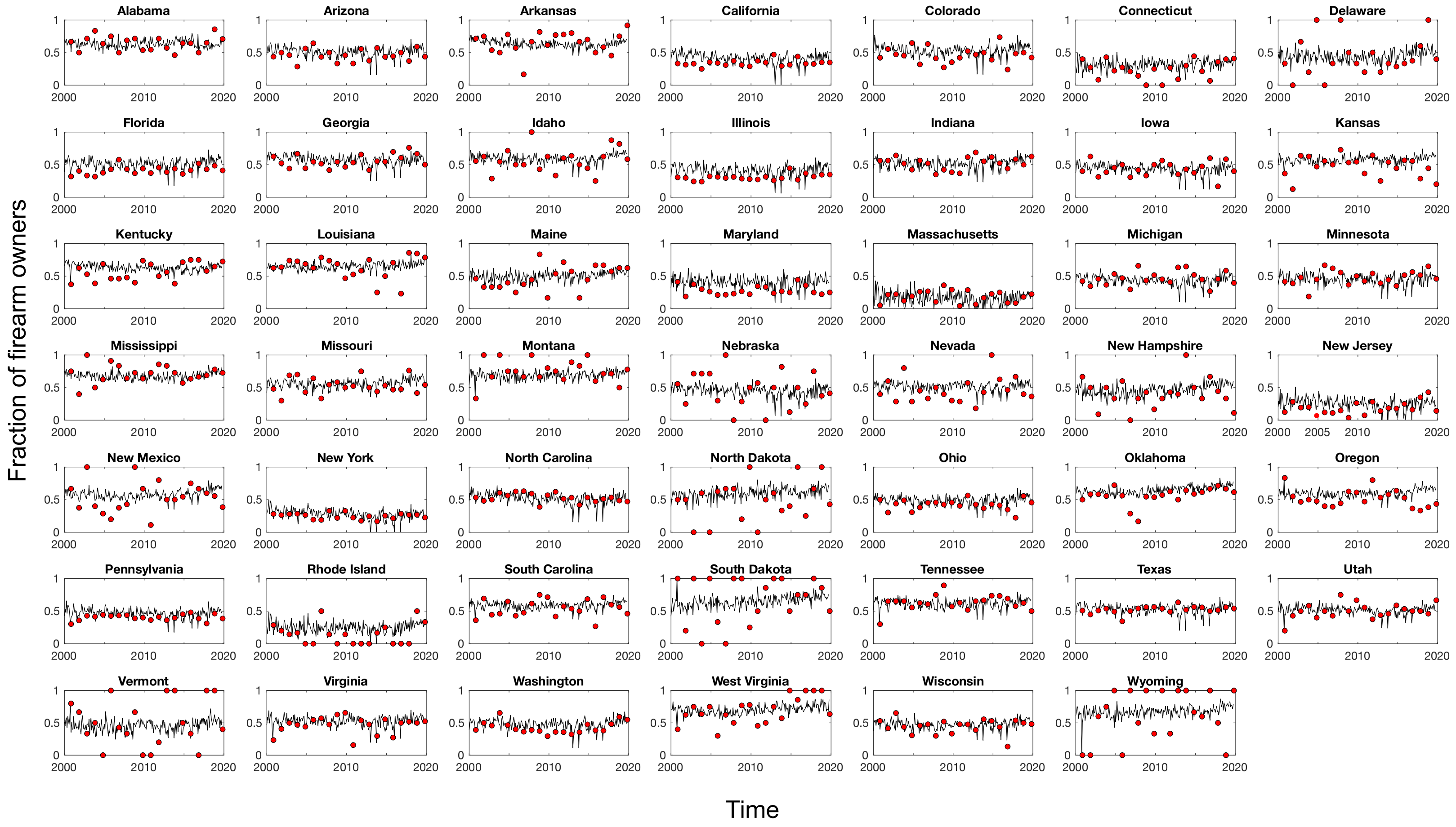

Figure S4: Predicted state-level firearm ownership. Each panel displays the model's output for monthly fraction of firearm owners in each U.S. state considered in the analysis. The red circles overlaying the time series reflect the fraction of firearm owners obtained from GPSS survey responses. Alaska and Hawaii were excluded from the analysis due to missing data.

$W_{i,j}$	ρ	τ	η	$\phi^{(1,\mathrm{H})}$	$\phi^{(1,\mathrm{L})}$	$\phi^{(2,\mathrm{H})}$	$\phi^{(2,\mathrm{L})}$	$\psi^{(1)}$	$\psi^{(2)}$	γ	$\alpha^{(1)}$	$\alpha^{(2)}$	σ^2
Null	N/A	0.0650<br>(2.0073)	N/A	14.9787<br>(1.9866)	33.1150<br>(7.0849)	0.6591<br>(7.7568)	0.4213<br>(7.5694)	N/A	N/A	0.0219<br>(0.5640)	0.1086<br>(3.4428)	0.0024<br>(2.1117)	0.0329
$\frac{1}{D_{i,j}}$	0.2120<br>(2.1290)	0.0111<br>(0.3378)	0.2037<br>(1.3012)	15.8757<br>(2.1169)	35.6671<br>(7.5810)	0.4956<br>(5.7229)	0.3035<br>(5.2556)	-49.1362<br>(-2.8931)	0.8756<br>(3.2641)	0.0058<br>(2.8418)	-0.3954<br>(-4.7000)	-0.3182<br>(-3.8803)	0.0314
A_j	-0.0190<br>(-0.0439)	0.0568<br>(1.8018)	0.0270<br>(0.0915)	17.8256<br>(2.3664)	34.2948<br>(7.4053)	0.6540<br>(7.8235)	0.4259<br>(7.6995)	-45.0485<br>(-2.2004)	-0.3368<br>(-1.0503)	0.3377<br>(0.5108)	0.4210<br>(0.6397)	0.0041<br>(2.0864)	0.0325
p_j	-0.0010<br>(-0.0038)	0.0555<br>(1.7301)	-0.6913<br>(-1.9739)	16.4106<br>(2.1853)	35.0488<br>(7.5007)	0.6302<br>(7.5426)	0.4233<br>(7.6791)	-67.0699<br>(-2.8422)	-0.1708<br>(-0.5053)	0.0081<br>(2.3780)	0.5318<br>(3.5903)	0.5981<br>(4.0656)	0.0323
$\frac{p_j}{A_j}$	0.0210<br>(1.4630)	0.0563<br>(1.7396)	-0.3133<br>(-1.9207)	16.8028<br>(2.2359)	34.8698<br>(7.4714)	0.6316<br>(7.4828)	0.4178<br>(7.5422)	-79.3382<br>(-3.4242)	0.0406<br>(0.1155)	0.0089<br>(3.4519)	0.2214<br>(1.4666)	0.2943<br>(1.9815)	0.0324
$\frac{p_j}{D_{i,j}}$	0.1600<br>(4.2194)	0.0034<br>(0.1064)	-0.0489<br>(-0.1738)	18.1607<br>(2.4782)	36.5966<br>(8.0076)	0.5285<br>(6.3866)	0.2742<br>(4.7925)	-70.2875<br>(-4.6749)	1.6014<br>(4.0111)	0.0104<br>(5.8702)	-0.6226<br>(-10.1421)	-0.5081<br>(-10.6513)	0.0310
$\frac{p_j}{D_{i,j}A_j}$	0.1760<br>(1.9553)	0.0155<br>(0.4762)	-0.0100<br>(-0.0714)	14.5327<br>(1.9608)	35.1491<br>(7.5903)	0.5176<br>(6.0526)	0.3183<br>(5.5524)	-38.0757<br>(-1.8346)	0.8851<br>(3.5212)	0.0068<br>(2.8018)	-0.2753<br>(-4.2243)	-0.2016<br>(-3.1926)	0.0313
GDP_j	-0.0010<br>(-0.0037)	0.0557<br>(1.7354)	-0.6909<br>(-1.9285)	16.3119<br>(2.1723)	35.0451<br>(7.4988)	0.6257<br>(7.4988)	0.4236<br>(7.6831)	-69.4314<br>(-2.9345)	-0.1163<br>(-0.3799)	0.4963<br>(3.1898)	0.5603<br>(3.6383)	0.0080<br>(2.3640)	0.0323
$\frac{GDP_j}{D_{i,j}}$	0.1330<br>(1.6031)	0.0011<br>(0.0342)	-0.1353<br>(-0.7012)	17.8700<br>(2.4393)	36.5014<br>(7.9754)	0.5354<br>(6.4673)	0.2741<br>(4.7887)	-69.9531<br>(-4.3995)	1.6713<br>(4.4609)	-0.6060<br>(-10.4091)	-0.4899<br>(-10.8460)	0.0115<br>(7.0143)	0.0309
$\frac{GDP_j}{A_{i,j}}$	0.0170<br>(1.4089)	0.0575<br>(1.7812)	-0.3111<br>(-1.9837)	16.8049<br>(2.2356)	34.8574<br>(7.4656)	0.6315<br>(7.4644)	0.4210<br>(7.6062)	-81.5104<br>(-3.5195)	0.1674<br>(0.5417)	0.1586<br>(1.2090)	0.2296<br>(1.8034)	0.0093<br>(3.6516)	0.0323
$\frac{GDP_j}{A_jD_{i,j}}$	0.1540<br>(4.6494)	0.0182<br>(0.5580)	-0.0411<br>(-0.3026)	14.5826<br>(1.9664)	34.9804<br>(7.5542)	0.5352<br>(6.2956)	0.3260<br>(5.7030)	-34.5256<br>(-1.7052)	0.9322<br>(4.3198)	-0.2876<br>(-4.3994)	-0.2097<br>(-3.3446)	0.0074<br>(3.2527)	0.0314
$B_{i,j}$	0.0570<br>(5.3576)	0.0339<br>(1.0439)	0.1188<br>(1.9000)	20.7151<br>(2.7070)	38.8034<br>(8.0946)	0.5866<br>(6.8340)	0.3500<br>(6.1780)	-25.2057<br>(-3.7354)	0.2072<br>(2.2947)	0.0033<br>(2.6990)	-0.0673<br>(-1.3607)	0.0298<br>(0.6144)	0.0320
$B_{i,j}p_j$	0.0400<br>(1.0093)	0.0360<br>(1.1092)	0.1402<br>(1.6833)	22.5310<br>(2.9529)	38.1605<br>(8.0844)	0.6224<br>(7.3597)	0.3391<br>(5.9250)	-34.3594<br>(-4.4866)	0.1590<br>(1.5393)	0.0039<br>(3.0682)	-0.0521<br>(-1.0860)	0.0783<br>(1.7655)	0.0320
$B_{i,j}GDP_j$	0.0390<br>(1.0030)	0.0357<br>(1.0985)	0.1435<br>(1.7325)	22.5302<br>(2.9541)	38.1463<br>(8.0873)	0.6228<br>(7.3648)	0.3391<br>(5.9284)	-34.5347<br>(-4.5214)	0.1564<br>(1.5209)	0.0040<br>(3.0765)	-0.0517<br>(-1.0749)	0.0793<br>(1.7735)	0.0320

Table S1: Exploring alternative spatial models. Results of econometric spatial model parameters upon calibration with different formulations of the spatial weight matrix W. The t-statistic signifying the difference of the estimated parameter from zero is denoted in the parentheses.

State	SSE	MSE	State	SSE	MSE
Alabama	0.4510	0.0226	Nebraska	1.9089	0.0954
Arizona	0.4385	0.0219	Nevada	0.9087	0.0454
Arkansas	0.6647	0.0332	New Hampshire	1.1838	0.0592
California	0.2967	0.0148	New Jersey	0.3612	0.0181
Colorado	0.4115	0.0206	New Mexico	1.1929	0.0592
Connecticut	0.4715	0.0236	New York	0.2002	0.0100
Delaware	1.6216	0.0811	North Carolina	0.3470	0.0173
Florida	0.2151	0.0108	North Dakota	2.0258	0.1013
Georgia	0.4114	0.0206	Ohio	0.1652	0.0083
Idaho	0.6780	0.0339	Oklahoma	0.4149	0.0207
Illinois	0.2012	0.0101	Oregon	0.4445	0.0222
Indiana	0.4450	0.0222	Pennsylvania	0.1703	0.0085
Iowa	0.5960	0.0298	Rhode Island	0.5536	0.0277
Kansas	0.7251	0.0363	South Carolina	0.4309	0.0215
Kentucky	0.4833	0.0242	South Dakota	2.7643	0.1382
Louisiana	0.4806	0.0240	Tennessee	0.5156	0.0258
Maine	0.5292	0.0265	Texas	0.3101	0.0155
Maryland	0.4596	0.0230	Utah	0.2846	0.0142
Massachusetts	0.4472	0.0224	Vermont	2.5937	0.1297
Michigan	0.6966	0.0348	Virginia	0.3948	0.0197
Minnesota	0.7136	0.0357	Washington	0.2351	0.0118
Mississippi	0.4534	0.0227	West Virginia	0.7062	0.0353
Missouri	0.3962	0.0198	Wisconsin	0.2886	0.0144
Montana	0.6400	0.0320	Wyoming	2.5167	0.1258

Table S2: Evaluation of the model's output. The model's output is compared against firearm ownership measured by the Gallup Poll Social Series Crime surveys through sum of squared errors (SSE) and mean of squared errors (MSE).

State	Firearm ownership	Background checks	Background checks per capita	Fraction of suicides with firearms
Alabama	0.2842	**0.0132**	**0.0131**	0.2661
Arizona	0.1989	0.2346	0.1929	0.3076
Arkansas	0.3228	**0.0405**	**0.0379**	0.2063
California	0.1140	0.0508	0.0521	0.3460
Colorado	0.1821	0.3032	0.2322	0.1894
Connecticut	**0.0154**	0.1583	0.1581	**0.0024**
Delaware	**0.0621**	0.2242	0.1989	**0.0010**
Florida	0.1886	0.4059	0.3666	0.3398
Georgia	0.2539	**0.0167**	**0.0142**	0.3139
Idaho	0.3364	0.1189	0.0920	0.0723
Illinois	0.0750	0.0638	0.0643	0.1650
Indiana	0.1981	0.2199	0.2090	0.1683
Iowa	0.1389	**0.0114**	**0.0108**	**0.0400**
Kansas	0.3526	0.1385	0.1330	0.1069
Kentucky	0.3394	**0.0426**	**0.0410**	0.1875
Louisiana	0.3889	**0.0352**	**0.0341**	0.2486
Main	0.1857	0.0977	0.0938	**0.0127**
Maryland	**0.0235**	**0.0076**	**0.0077**	0.0589
Massachusetts	**0.0010**	0.3499	0.3427	**0.0025**
Michigan	0.1467	0.1158	0.1158	0.2737
Minnesota	0.0975	0.0861	0.0776	0.1091
Mississippi	0.3401	**0.0127**	**0.0125**	0.2119
Missouri	0.2982	0.2300	0.2198	0.2241
Montana	0.3484	0.0999	0.0865	**0.0433**
Nebraska	0.1184	**0.0152**	**0.0140**	**0.0082**
Nevada	0.2566	0.1720	0.1433	0.1456
New Hampshire	0.0981	0.3308	0.3207	**0.0021**
New Jersey	**0.0024**	0.5076	0.5025	**0.0330**
New Mexico	0.3579	0.2428	0.2239	0.0768
New York	**0.0200**	0.3205	0.3182	0.1422
North Carolina	0.1583	**0.0123**	**0.0098**	0.2797
North Dakota	0.2261	0.1277	0.1077	**0.0017**
Ohio	0.1909	0.1698	0.1679	0.3213
Oklahoma	0.4330	0.1313	0.1227	0.2298
Oregon	0.3625	0.2504	0.2147	0.2198
Pennsylvania	0.1523	0.2783	0.2747	0.3018
Rhode Island	**0.0017**	0.3368	0.3355	**0.0010**
South Carolina	0.3369	0.0502	**0.0414**	0.1802
South Dakota	0.3011	0.1425	0.1234	**0.0010**
Tennessee	0.3110	0.1167	0.1036	0.3289
Texas	0.2335	0.0929	0.0749	0.4433
Utah	0.2510	**0.0215**	**0.0163**	0.0683
Vermont	0.0709	0.2077	0.2053	**0.0010**
Virginia	0.1664	0.2506	0.2290	0.2585
Washington	0.1646	0.3464	0.3116	0.2496
West Virginia	0.3771	0.0915	0.0935	0.1375
Wisconsin	0.1773	0.1831	0.1739	0.1591
Wyoming	0.2410	0.1043	0.0962	**0.0061**

Table S3: Stationarity of State-level time series.Reported p-values for the augmented Dickey-Fuller test, applied on raw data between January 2000 and December 2017. Values in bold represent a stationarity at a significant level of 0.05.

Date	Event	Date	Event
12/26/00	Edgewater Technology shooting, MA	4/21/13	Pinewood Village Apartments shooting, WA $*$
2/5/01	Navistar International shooting, IL	6/7/13	Santa Monica College shooting, CA $*$
7/8/03	Lockheed Martin shooting, MS	7/26/13	Todel Apartments shooting, FL
12/8/04	Damageplan show shooting, OH	9/16/2013	The Washington Navy Yard shooting, DC
3/12/05	Living Church of God shooting, WI	2/20/14	Cedarville Rancheria Tribal Office shooting, CA $*$
3/21/05	Red Lake Indian reservation shooting, MN	4/3/14	Fort Hood shooting II, TX $*$
1/30/06	Postal facility shooting, CA $*$	5/23/14	Santa Barbara County shooting, CA $*$
3/25/06	Capitol Hill shooting, WA $*$	10/24/14	Marysville-Pilchuck High School shooting, WA $*$
10/2/06	Amish School shooting, PA	6/11/15	Trestle Trail bridge shooting, WI
2/12/07	Trolley Square shooting, UT	6/17/15	Charleston Church Shooting, SC
4/16/07	Virginia Tech shooting, VA	7/16/15	Chattanooga military recruitment center, TN
10/7/07	Crandon duplex shooting, WI	10/1/15	Umpqua Community College shooting, OR $*$
12/5/07	Westroads Mall shooting, NE	10/31/15	Colorado Springs shooting rampage, CO
2/7/08	City council shooting, MO	11/27/15	Planned Parenthood clinic, CO
2/14/08	Northern Illinois University shooting, IL	12/2/15	Inland Regional Center shooting, CA $*$
6/25/08	Atlantis Plastics shooting, KY	2/20/16	Cracker Barrel shooting, MI
3/29/09	Carthage nursing home shooting, NC	2/25/16	Excel Industries mass shooting, KS
4/3/09	Immigration services center shooting, NY	6/12/16	Pulse nightclub shooting, FL
11/5/09	Army processing center shooting, TX $*$	7/7/16	Police protest march shooting, TX $*$
11/29/09	Pierce County coffee shop shooting, WA $*$	7/17/16	Baton Rouge police shooting, LO
8/3/10	Hartford Beer Distributors shooting, CT	9/23/16	Cascades Mall Macy's shooting, WA $*$
1/8/11	Safeway parking lot shooting, AZ $*$	1/6/17	Fort Lauderdale airport shooting, FL
9/6/11	IHOP shooting, NV $*$	4/18/17	Fresno downtown shooting, CA $*$
10/12/11	Salon Meritage shooting, CA $*$	5/12/17	Rural Ohio nursing home shooting, OH
2/21/12	Su Jung Health Sauna shooting, GA	6/5/17	Florida awning manufacturer shooting, FL
4/2/12	Oikos University shooting, CA $*$	6/7/17	Pennsylvania supermarket shooting, PA
5/30/12	Café Racer shooting, WA $*$	6/14/17	San Francisco UPS shooting, CA $*$
7/20/12	Century 16 movie theater shooting, CO	10/1/17	Las Vegas Strip massacre, NV $*$
8/5/12	Sikh temple of Wisconsin shooting, WI	10/18/17	Edgewood business park shooting, MD
9/27/12	Accent Signage Systems shooting, MN	11/1/17	Walmart shooting in suburban Denver, CO
12/14/12	Sandy Hook Elementary School shooting, CT	11/5/17	First Baptist Church shooting, TX $*$
3/13/13	Mohawk Valley shootings, NY	11/14/17	Rancho Tehama shooting spree, CA $*$

Table S4: Mass shootings in the U.S. between January 2000 and December 2017. The data were was obtained from Mother Jones and exclude the U.S. Territories. $*$ denotes events that took place in the West and Southwest (24 out of 64 events in total).

	Firearm ownership	Mass shootings	Media output
Firearm ownership	–	0.0464 $*$ (0.0128)	0.0579 $*$ (0.0026)
Mass shootings	0.0098 (0.5993)	–	0.0137 (0.4665)
Media output	0.0302 $\circ$ (0.0755)	0.0231 (0.1831)	–

Table S5: Causal analysis in the absence of τ and η. Transfer entropy was computed between firearm ownership, mass shootings, and media output, where the time series for firearm ownership was estimated from model neglecting τ and η. Rows are sources and columns are targets. The numbers in parentheses denote the p-value obtained from a permutation test.

.

MO to BC, conditioned on MS (MO→ BC|MS)

(a) (b)

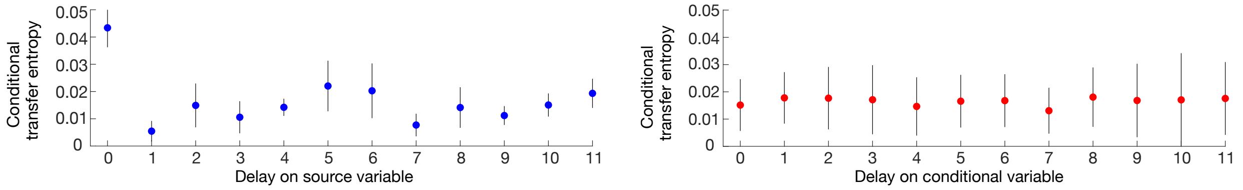

FO to MS, conditioned on MO (FO→ MS|MO)

(c) (d)

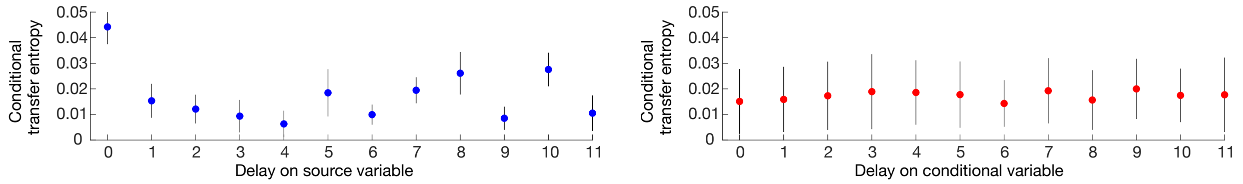

FO to MO, conditioned on MS (FO→ MO|MS)

(e) (f)

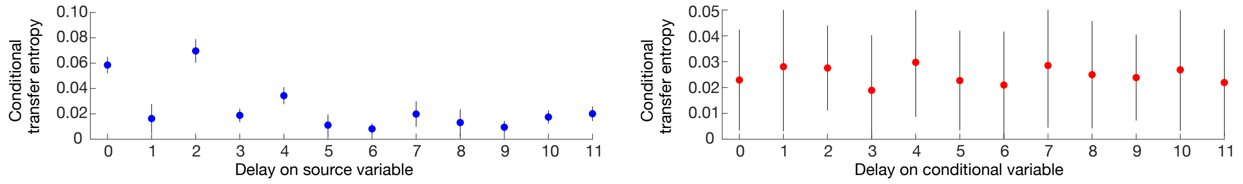

MO to FO, conditioned on MS (MO→ FO|MS)

(g) (h)

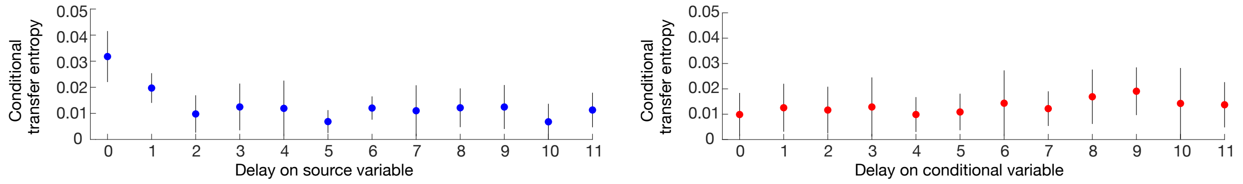

Figure S5: Delay analysis for causal links. Conditional transfer entropy was computed for national level time series with delays on the source variable time series (a,c,e,g) or the conditional variable (b,d,f,h). The round markers represent the mean of the delay, computed over the 12 delays of the source variable or conditional variable. The vertical bars represent $\pm$ one standard deviation.

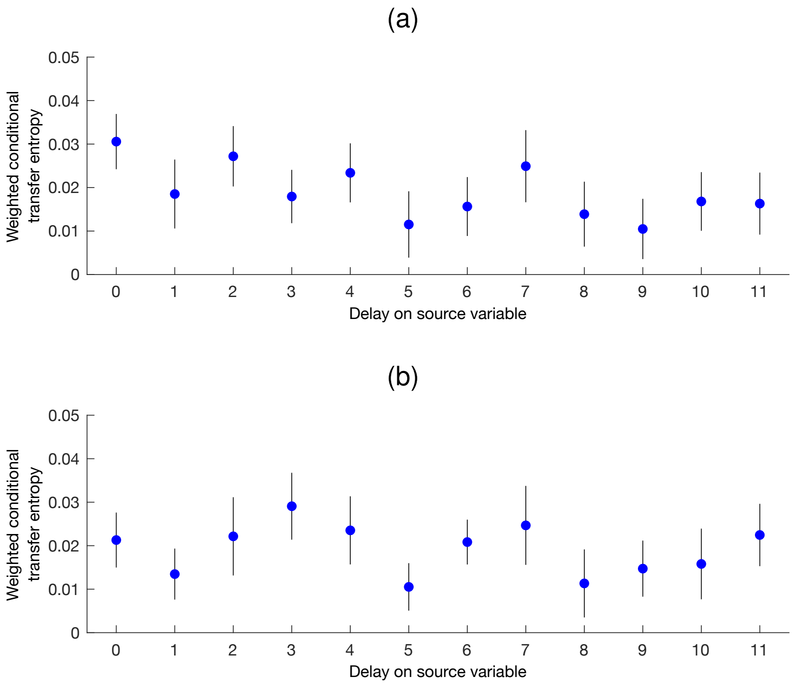

Figure S6: Delay analysis in permissive and restrictive states. Delayed conditional transfer entropy from FO to MO was computed separately for permissive states (a) and restrictive states (b). The round markers represent the mean value of transfer entropy across states, weighted by their population size. The vertical bars represent $\pm$ one standard deviation.

	Firearm ownership	Mass shootings	Media output
Firearm ownership	–	0.0123 (0.4819)	0.0116 (0.5284)
Mass shootings	0.0144 (0.4056)	–	0.0057 (0.8157)
Media output	0.0240 (0.1540)	0.0123 (0.4877)	–

Table S6: Causal analysis in the absence of spatial associations. Transfer entropy was computed between firearm ownership, mass shootings, and media output, where the time series for firearm ownership was generated from model neglecting spatial interactions ($W = 0$). Rows are sources and columns are targets. The numbers in parentheses denote the p-value obtained from a permutation test.

.
