# Supplementary material for: A spatiotemporal model of firearm ownership in the United States
Source: Patterns (N Y). 2022 Jun 29;3(8):100546. doi: 10.1016/j.patter.2022.100546 (PMC9403408; doi:10.1016/j.patter.2022.100546)
Supplement: Document S2. Article plus supplemental information [file mmc3.pdf]

Article

Patterns

A spatiotemporal model of firearm ownership in the United States

Graphical abstract

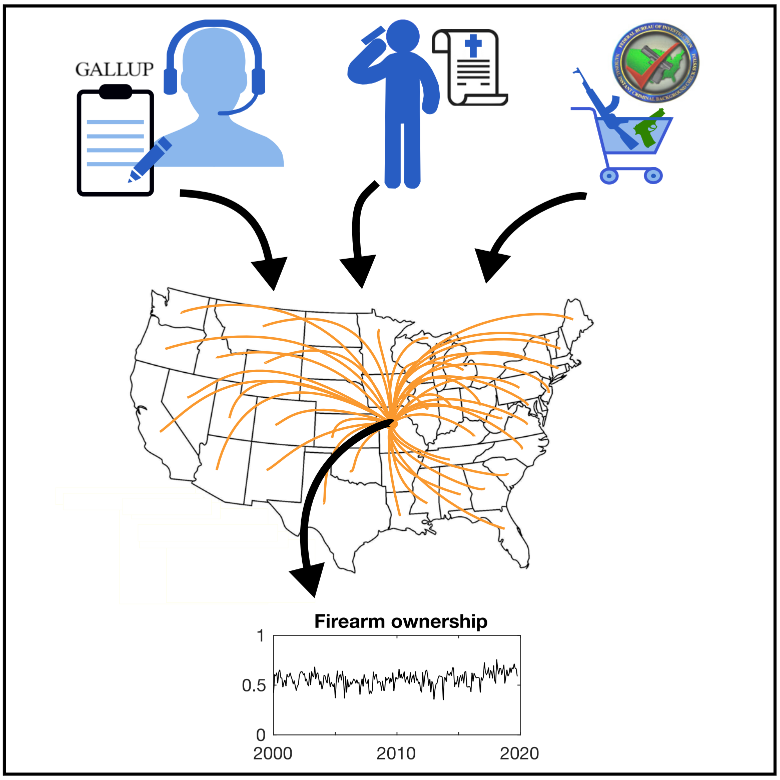

Highlights

- A spatiotemporal model of firearm prevalence in the United States is created
- The econometric model predicts firearm ownership in every state for every month
- Information theory is used to detail causal links related to firearm prevalence
- The media can influence firearm prevalence, which in turn moderates mass shootings

Authors

Roni Barak-Ventura,
Manuel Ruiz Marín, Maurizio Porfiri

Correspondence

mporfiri@nyu.edu

In brief

In the absence of a national firearm registry, would it be possible to estimate firearm ownership with high spatial and temporal resolution? A spatial econometric model is developed to predict firearm ownership from data on background checks and suicides committed with a firearm. The model's output is utilized in an information-theoretic analysis of causal relationships between firearm prevalence, media output on firearm regulations, and mass shootings. The analysis reveals new links that are masked by use of either data source individually.

Barak-Ventura et al., 2022, Patterns *3*, 100546
August 12, 2022 © 2022 The Author(s).
https://doi.org/10.1016/j.patter.2022.100546

CellPress

Patterns

CellPress
OPEN ACCESS

Article

A spatiotemporal model of firearm ownership in the United States

Roni Barak-Ventura,[1,2] Manuel Ruiz Marín,[3,4] and Maurizio Porfiri[1,2,5,6,*]
[1]Department of Mechanical and Aerospace Engineering, New York University Tandon School of Engineering, Brooklyn, NY 11201, USA
[2]Center for Urban Science and Progress, New York University Tandon School of Engineering, Brooklyn, NY 11201, USA
[3]Department of Quantitative Methods, Law and Modern Languages, Technical University of Cartagena, Cartagena, 30201 Murcia, Spain
[4]Murcia Bio-Health Institute (IMIB-Arrixaca), Health Science Campus, Cartagena, 30120 Murcia, Spain
[5]Department of Biomedical Engineering, New York University Tandon School of Engineering, Brooklyn, NY 11201, USA
[6]Lead contact
*Correspondence: mporfiri@nyu.edu
https://doi.org/10.1016/j.patter.2022.100546

THE BIGGER PICTURE Firearm violence is a major public health crisis in the United States, where more than 200 people sustain a nonfatal firearm injury and more than 100 people die from it every day. Despite these unsettling figures, scientific research on firearm-related harm significantly lags behind because spatially and temporally resolved data on firearm ownership are unavailable. This paper presents a spatiotemporal model that predicts firearm prevalence at the resolutions of one state and one month from the numbers of background checks and suicides committed with a firearm. Drawing on principles from econometrics, the model also accounts for interactions between states. The model's output is challenged in causal analysis, which uncovers unprecedented associations between firearm prevalence, media output on firearm regulations, and mass shootings.

1 2 3 4 5 **Development/Pre-production:** Data science output has been rolled out/validated across multiple domains/problems

SUMMARY

Firearm injury is a major public health crisis in the United States, where more than 200 people sustain a nonfatal firearm injury and more than 100 people die from it every day. To formulate policy that minimizes firearm-related harms, legislators must have access to spatially resolved firearm possession rates. Here, we create a spatiotemporal econometric model that estimates monthly state-level firearm ownership from two cogent proxies (background checks per capita and fraction of suicides committed with a firearm). From calibration on yearly survey data that assess ownership, we find that both proxies have predictive value in estimation of firearm ownership and that interactions between states cannot be neglected. We demonstrate use of the model in the study of relationships between media coverage, mass shootings, and firearm ownership, uncovering causal associations that are masked by the use of the proxies individually.

INTRODUCTION

Firearm violence is a serious public health threat in the United States. Every year, more than 67,000 people in the United States are injured by firearms.[1] The costs associated with their initial hospitalization alone amount to $750 million per year,[2] and long-term medical care and productivity loss are estimated to tally above $88 billion.[3] Firearm-related death statistics are also exceptionally grim in the United States. In 2018, the National Center for Health Statistics has reported nearly 40,000 deaths due to firearm-related violence in the United States, amounting to 109 deaths per day and surpassing the number of deaths due to motor vehicle accidents.[2,4]

Accessibility to firearms in the United States has been repeatedly correlated with firearm violence, where states with greater firearm possession rates experience a higher risk of suicides, homicides, and assaults with firearms.[5–8] Despite these findings, most Americans do not welcome laws that restrict firearm purchases and ownership.[9] In a 2013 survey by the Pew Research Center (PRC), 58% of firearm owners and non-owners expressed concern that new firearm laws will make it more difficult for people to protect their homes and families.[10] In fact, many

This is an open access article under the CC BY license (http://creativecommons.org/licenses/by/4.0/).

Americans hold the belief that ubiquitous firearms could confer protection to their community.[9] In a 2019 study conducted by the PRC, 67% of firearm owners cited protection as the main reason for owning a firearm.[11] According to another poll by the National Broadcasting Company (NBC) in 2018, 58% of American adults thought that firearms increase safety by allowing law-abiding citizens to protect themselves.[12]

Thus, policy-makers are faced with an exceptional challenge: reducing harm caused by firearms while maintaining citizens' right to bear arms and protect themselves.[13] To meticulously study how access to firearms is associated with different outcomes of harm, it is imperative that policymakers gain access to accurate, highly resolved data on firearm possession. Unfortunately, such measurements are presently unavailable as no comprehensive national firearm ownership registry or other reliable record of firearm acquisition exists. Instead, the requirement to register firearms varies on a state-to-state basis.[14]

In the absence of national firearm registries and in light of the strong opposition to map firearms to owners, anonymous survey instruments are the method of choice to measure firearm ownership. However, an accurate estimate of firearm ownership on a state level and its variation over time requires a high response rate across geographical regions and demographic populations at a high temporal resolution. Three surveys that assess firearm ownership in American households are highly regarded among researchers: Behavioral Risk Factor Surveillance System (BRFSS) surveys, the PRC surveys, and Gallup Poll Social Series (GPSS).

The BRFSS is a system of health-related telephone surveys conducted by the National Center for Chronic Disease Prevention and Health Promotion (NCCDPHP) at the Centers for Disease Control and Prevention (CDC).[15] Established in 1984, the system methodically interviewed 400,000 adult across the 50 states to inquire about their health-related risk behaviors, chronic health conditions, and use of preventive services. Data from the BRFSS are only available up to 2014, and data on firearm ownership were only collected in 3 of the 20 years (2001, 2002, and 2004). Therefore, BRFSS data cannot be used to reliably study temporal processes involving firearm ownership.

A second survey that measures firearm ownership is conducted by the PRC. The PRC began administering surveys in the early 1990s, focusing primarily on United States policy and politics, with questions regarding firearm ownership available through the American Trends Panel.[16] The PRC surveys are conducted online every couple of months (called "waves"), where the same respondents may participate. Therefore, the data collected by the PRC could illustrate how public opinion and behavior change over time. The PRC surveys are designed to be nationally representative, with more than 10,000 adults selected randomly across the entire country in each survey. However, the PRC data are not optimal for study of firearm ownership because questions about firearm ownership are administered sparsely and at irregular intervals, and in 2013, the PRC changed the wording for the question inquiring about firearm ownership, likely introducing a bias in the years thereafter.[17] Therefore, the data obtained from the PRC may be context specific and yield inconsistent results for longitudinal assessment of firearm ownership.

The third survey instrument, the GPSS, was designed to monitor United States adults' views on numerous social, economic, and political topics.[18] The GPSS has operated continuously since the 1930s and is an excellent source for generational studies. Survey topics are arranged thematically across 12 surveys, each administered one month a year. The crime survey series, which assesses firearm ownership among other issues, was conducted consistently in October from 2000 until 2021. The GPSS interviews a minimum of 1,000 United States adults in all 50 states through landline and cellphone numbers. The greatest limitation of GPSS data is that they are not designed to be representative of populations in individual states; for some years, responses from only one resident in a state were obtained. Therefore, inference of state-level firearm ownership in less densely populated states is suboptimal using GPSS data.

Several alternative measures have been proposed to estimate firearm prevalence in the United States. In particular, proxies derived from administrative data collected by government agencies are available at the state level (and even county level) over a long time period. One such measure is background checks, collected by the Federal Bureau of Investigation's National Instant Criminal Background Check System (NICS).[19] The NICS was established in November 1998, following legislation of the Brady Handgun Violence Prevention Act, which conditioned firearm purchases on approval of federal background checks.[20] Using the NICS, authorized firearm vendors submit a background check request to determine the eligibility of prospective buyers to purchase firearms. Background check data are available on the NICS at the state level on a monthly resolution, also specifying the type of transactions performed, including sales, pre-pawns, rentals, and redemptions. More recently, the Federal Bureau of Investigation (FBI) has released the daily number of background checks on a national level, allowing more granular analysis of firearm acquisition across states. Due to these features, background checks have been used extensively in previous research to approximate firearm acquisitions in United States states.[21] However, the number of background checks only serves as an approximation of the number of firearms that are actually purchased every month.[21,22] Background checks do not always realize into an acquisition, and they do not capture illegal firearm sales.[22] Conversely, private-party sales and firearm show sales may not yield a background check because only licensed federal dealers are required to do so.[21]

Another measure that is widely used among firearm policy researchers is the number of suicides committed with firearms. Data on suicides and their underlying causes can be readily obtained from the CDC's Wonder database.[4] Wonder's national mortality and population database is managed by the National Center for Health Statistics based on death certificates for United States residents. It fuels multi-faceted public health studies, accounting for many demographic aspects surrounding harmful factors. In various correlation analyses, the fraction of suicides committed with firearms was heralded as the best proxy for firearm ownership in the United States.[7,8,17,23] However, similar to background checks, this measure is only an approximation of firearm possession. The means by which suicides are committed is not always driven by accessibility to firearms or lack thereof. For example, women tend to choose less violent methods, such as drugs and carbon monoxide poisoning, even

CellPress
OPEN ACCESS

when they have access to firearms.[24] Self-inflicted harm could involve some social trends,[25,26] which will determine the relative proportion of suicides that are committed with firearms.

Additional empirical measures have been proposed in the past to better approximate firearm possession rates, including the percentage of homicides committed with firearms,[6,23,27] the fraction of firearm-armed robberies,[6,28] the number of hunting licenses per capita,[6,17] and the fatal firearm accident rate,[6,23] although support for the validity of these measures is mixed among researchers.[5,29,30] Efforts were also made to develop composite indicators that account for multiple proxy measurements simultaneously. For example, Cook[31] proposed a 2-item measure containing the number of suicides with firearms and homicides with firearms, and Kleck and Patterson[30] proposed a 5-item factor. Most recently, Schell et al.[17] combined survey measures with some commonly used proxies to estimate state-level firearm ownership in an accurate manner. In particular, the group used multi-level regression with post-stratification to derive an integrative measure of firearm ownership from surveys. This approach would emphasize estimates for subpopulations even when they are not equally represented.[17] Then, the authors created a structural equation model to incorporate proxy indicators of firearm ownership. The resulting model was compared with the individual survey instruments, demonstrating strong correlations with each.

A key limitation in formulation of proxies of firearm prevalence is associated with methodology, the vast majority of the aforementioned measures was grounded in simple correlational analyses only. Because rates for firearm-related violence appear to increase over time, correlations will yield faulty claims without pre-processing and detrending of time series.[32] Correlation-based schemes generally do not account for interactions between states. Most studies aggregate the measure counts within states and do not consider interference between states or spillover effects.[33] There is mounting evidence that such ecological study designs, where one assumes that spatial units are independent and do not affect outcomes in other units, are not appropriate for studying state policies in the United States because such interactions exist.[33–36] Therefore, a spatial approach that accounts for geographic interactions may be more suitable to quantify firearm ownership.

Spatial econometrics is a promising means to empirically support firearm policies. Spatial econometrics emerged in the 1970s to model the dynamic growth and decline of European cities.[37] Since then, its use has extended to study processes in labor economics, transportation, agriculture, and environmental science.[37] Unlike time series, which vary along a single axis (time), spatial data lack uniform organization and could vary in multiple directions.[38] Therefore, spatial econometric models aim to capture spatial interactions (also known as spatial autocorrelation) and structure (also known as spatial heterogeneity) in cross-sectional data[37,38] through five guiding principles: (1) there exists a spatial interdependence between units, (2) spatial relations are asymmetric, (3) explanatory factors located in other spaces can have direct and indirect influence, (4) *ex post* and *ex ante* interactions must be distinguished, and (5) topology needs to be explicitly accounted for.[37]

Here, we aim to develop a spatiotemporal model that predicts state-level firearm ownership on a monthly resolution. We borrow methodologies from spatial econometrics to model interactions between states while accounting for multiple firearm prevalence measures simultaneously. The model integrates data from multiple proxies so that it predicts firearm ownership from the number of background checks per capita and the fraction of suicides committed with firearms, with calibration on GPSS survey data on firearm ownership. In this manner, the model capitalizes on the advantages of existing data sources while mitigating the aforementioned limitations. We detail the calibration results to elucidate the role of each proxy in predicting firearm ownership and to unravel spatial processes that might take place between states. Finally, we demonstrate the value of the integrative model in the study of determinants and consequences of firearm ownership. Specifically, we revisit the conclusions of our previous work on causal interactions within a triad composed of firearm prevalence, mass shootings, and media output.[22] We show that, by merging different proxies into a unified model, we are able to detect causal processes that otherwise remain hidden.

RESULTS

Spatiotemporal model

The main contribution of this study is a spatiotemporal model to predict firearm prevalence on a state level. The model was derived from the spatial Durbin model (SDM), which accounts for interactions between states.[39] In its simplest form, an SDM for n observations (United States states in our case) is structured as

$$Y = \rho WY + \beta X + \theta WX + \alpha i_n + \varepsilon \quad \text{(Equation 1)}$$

where Y is an n-dimensional vector containing the dependent variable (firearm prevalence we aim to predict), and X is an n-dimensional vector containing the independent variable (the proxy used to measure firearm prevalence; that is, background checks per capita or fraction of suicides with firearms). In Equation (1), W is an $n \times n$ spatial weight matrix that quantifies the interactions between the n units, ρ is a scalar parameter that modulates the autoregressive process of the dependent variable, β is a scalar associated with the independent variables, θ is a scalar that modulates the spatial interaction of the independent variables, i_n is a vector of ones, α is a weighting scalar, and ε is an n-dimensional vector of n independent noise terms with zero mean and variance σ^2. W adds a weighted sum of Y and X from all spatial units as input to an observation of a certain spatial unit. In this manner, the dependent variable in a state is not predicted merely through a linear combination of the same state's independent variables. In the absence of spatial processes, the SDM reduces to an ordinary linear model $Y = \beta X + \alpha i_n + \varepsilon$, where β is the slope and α is the intercept.

To model processes that exhibit spatial and temporal variations, Elhorst[40] expanded the classical SDM toward a first-order autoregressive distributed lag model with spatial and temporal processes, expressed as

$$\begin{aligned} Y_t = {} & \rho WY_t + \tau Y_{t-1} + \eta WY_{t-1} + \beta X_t + \theta WX_t + \phi X_{t-1} \\ & + \psi WX_{t-1} + \alpha i_n + \varepsilon. \end{aligned} \quad \text{(Equation 2)}$$

In this specification, Y_t contains observations of the dependent variable in each spatial unit at different points in time. In this vein, Y_{t-1} contains observations of the dependent variable in each spatial unit at the serially preceding points in time. The scalars τ and ϕ modulate the memory effects of the dependent and independent variables, respectively. Similarly, scalars η and ψ modulate the memory of the spatial interaction for the dependent and independent variables, respectively.

We considered an extension of the Elhorst[40] model in Equation (2) to account for two independent, co-evolving processes (background checks per capita and fraction of suicides committed with firearms) and for the different time resolutions at which the dependent and independent variables are sampled (yearly versus monthly):

$$Y_m = \rho W_m Y_m + \tau Y_{m-12} + \eta W_m Y_{m-12} + \phi^{(1)} X^{(1)}_{m-1} + \phi^{(2)} X^{(2)}_{m-1} + \psi^{(1)} W_m X^{(1)}_{m-1} + \psi^{(2)} W_m X^{(2)}_{m-1} + \gamma d_n + \alpha i_n + \varepsilon. \quad \text{(Equation 3)}$$

In this model, a superscript of (1) refers to background checks per capita, and a superscript of (2) corresponds to the fraction of suicides committed with firearms. The subscript m represents a month in which the measurement was made, so that $m-12$ denotes an observation made in the same month in the previous year (12 months prior), and $m-1$ represents a measurement from the previous month. For example, should Y_m describe firearm prevalence measurements for every state in October 2004, then Y_{m-12} would represent the corresponding firearm prevalence in October of 2003 and $X^{(1)}_{m-1}$ background checks per capita in September of 2004. For completeness, we assumed the weight matrix to be time dependent. Finally, we introduced parameter γ and n-dimensional vector of dummy variables d_n, containing a unique integer in all of its entries for each year; the term γd_n would capture a linear time trend across years.

Our approach relied on survey responses as a direct measure of Y_m. During calibration, low response rates in less densely populated states would yield erroneous estimates of firearm ownership and, in return, would undermine maximum likelihood estimation. For example, in 2000, only one GPSS respondent was from Wyoming, and they reported no firearms in their possession, leading to the inference of 0 firearm ownership in that state that year. To mitigate miscalibration because of such inferences, we split the explicative variables in Equation (3) into two, based on the response rate (high or low),

$$Y_m = \rho W_m Y_m + \tau Y_{m-12} + \eta W_m Y_{m-12} + \phi^{(1,H)} X^{(1,H)}_{m-1} + \phi^{(1,L)} X^{(1,L)}_{m-1} + \phi^{(2,H)} X^{(2,H)}_{m-1} + \phi^{(2,L)} X^{(2,L)}_{m-1} + \psi^{(1)} W_{m-1} X^{(1)}_{m-1} + \psi^{(2)} W_{m-1} X^{(2)}_{m-1} + \gamma d_n + \alpha^{(H)} i^{(H)}_n + \alpha^{(L)} i^{(L)}_n + \varepsilon. \quad \text{(Equation 4)}$$

where vectors with a superscript H (high) include entries for states that had more than 10 respondents across all years and zeros otherwise, and vectors with a superscript L (low) contain entries for states that at least in one year had less than 10 respondents, and zero otherwise. Because of this split, two separate parameters would be estimated for ϕ^1, ϕ^2, and α during calibration, one for high-response states and another for low-response states.

Weight matrix of the model

W_m is an $n \times n$ matrix describing the spatial arrangement of the units in the sample; by definition, each of its elements is positive, and each of its row sums is 1. The spatial weight matrix is a key element in spatial models, and its construction is paramount to an SDM.[38,41]

We wished to account for the distance and population size of other states in our model. Thus, we formulated a W_m matrix so that closer and more populated states exert greater influence;[33] more specifically, the general off-diagonal i,j entry of W_m is

$$(W_m)_{i,j} = \frac{(p_m)_j}{(K_m)_i (D)_{i,j}} \quad \text{(Equation 5)}$$

where $(p_m)_j$ is the population size in state j in month m, $(D)_{i,j}$ is the distance between the geographical centroids of states i and j, and $(K_m)_i = \sum_{j=1, j\neq i}^{n} (p_m)_j / (D)_{i,j}$ is a row-normalizing factor. The diagonal entries are zero. Because census data on state population are only available on a yearly basis, W_m is constant for each year. Alternative forms of W were also examined for completeness, as presented in the supplemental information.

State-level data

State-level data were collected for our variables of interest: background checks (Figure 1A), background checks per capita (Figure 1B), and fraction of suicides committed with firearms (Figure 1C). Data on firearm ownership and background checks were missing for Alaska and Hawaii, respectively. Therefore, these states were excluded from the analysis, and only $n = 48$ states were considered.

Monthly data were collected between January 2000 and December 2019 on background check, background checks per capita, and fraction of suicides committed with firearms. Each of these datasets contained a total of 11,520 entries; Figures S1–S3 present those time series in each state. Firearm ownership data were only available on a yearly resolution from October 2000 to December 2019, amounting to a total of 960 recordings.

Model calibration and inference

Parameters ρ, τ, η, $\phi^{(1)}$, $\phi^{(2)}$, $\psi^{(1)}$, $\psi^{(2)}$, γ, α, and σ^2 were estimated using maximum likelihood, following LeSage and Pace (Table 1).[42] In maximum likelihood estimation, parameter values are determined by defining a likelihood function for the sample's probability distribution (GPSS reports for firearm ownership in our case) and computing the maximum of the function's natural logarithm. In our model, ρ, τ, and η's estimated values were 0.1600, 0.0034, and -0.0489, respectively; the estimated values of τ and η were indistinguishable from zero ($t = 0.1064$ and $t = -0.1738$, respectively), and ρ was different from zero ($t = 4.2194$). For background checks per capita, coefficients $\phi^{(1,H)}$ and $\phi^{(1,L)}$ had means of 18.1607 and 36.5966, respectively, and were significantly different than zero ($t = 2.4782$ and $t = 8.0076$, respectively). Similarly, coefficient $\psi^{(1)}$ was estimated at -70.2875 and was considered non-negligible ($t = -4.6749$). For the fraction of suicides with firearms, $\phi^{(2,H)}$ and $\phi^{(2,L)}$ assumed values of 0.5285 and 0.2742, respectively. Both coefficients were significantly different

CellPress
OPEN ACCESS

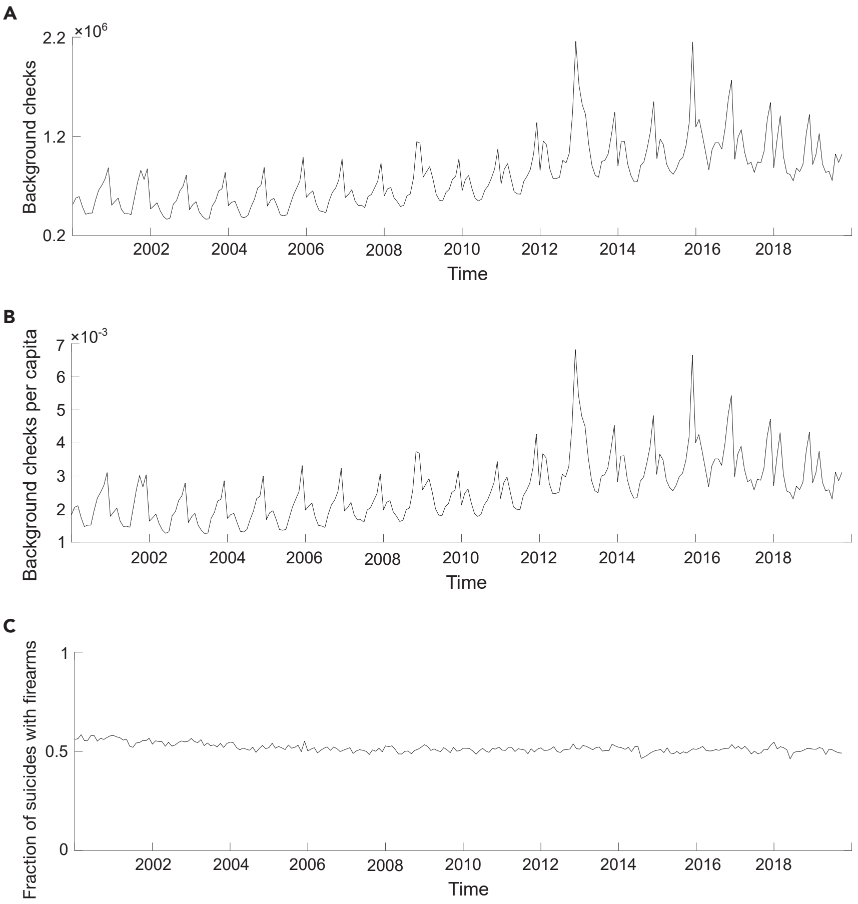

Figure 1. Time series for proxies of firearm ownership on a national level

(A–C) Time series between January 2000 and December 2019 for national-level background checks (A), background checks per capita (B), and fraction of suicides committed with firearms (C).

from zero ($t = 6.3866$ and $t = 4.7925$, respectively). Parameter ψ^2 was estimated at 1.6014 and was also different from zero ($t = 4.0111$). Finally, the intercepts α^H and α^L and linear time trend coefficient γ were estimated to be -0.6226, -0.5081, and 0.0104, respectively; all three were significantly different from zero ($t = -10.1421$, $t = -10.6513$, and $t = 5.8702$, respectively).

The calibrated parameters reflect a model whose spatial weight matrix encapsulates the strength of interactions between states based on their population size and distance. The elements of this matrix could include additional variables, such as states' geographical area, gross domestic product, and shared borders. In Table S1, we present the calibrated parameters for alternative models, where W contains these variables, as well as a null model without spatial interactions between states ($W = 0$). The results indicate that states' population size and distance minimize noise variance within an autoregressive model.

The model was calibrated once for all 20 years. Given the calibrated model parameters, we inferred state-level firearm prevalence on a monthly resolution for the 48 states under consideration. We specified the values obtained from 2000 (the first year when GPSS data were available) in each state as initial values for firearm ownership in the months of January–December 1999. Moving forward every month from January 2000, we computed firearm ownership in an iterative manner by plugging monthly values we collected on background checks per capita and fraction of suicides with firearms into Equation (4). To avoid drifting of the model for an extended prediction, we predict October data Y_m utilizing the survey data from the previous October, Y_{m-12} ($m = 22, 34, 46, \ldots, 238$). For the prediction of Y_m in the remaining months of the year, we use the model's output from the same month in the previous year, Y_{m-12}. We set the noise to $\sigma^2 = 0$ so that the inference is effectively for the mean value of firearm ownership.

Inferences were obtained on a national level as well. For each monthly entry, national background checks per capita were computed by aggregating the number of background checks across states and dividing the total by the population size in the 48 states in that year. Similarly, the national fraction of suicides committed with firearms was calculated by summing the monthly number of suicides by firearms across states and dividing by the total number of suicides. By iteratively plugging those monthly values into the model, we obtained firearm ownership on a national level. Alaska, the District of Columbia, Hawaii, and the five United States territories were excluded from national-level computations because of missing data. All variables were considered in our inference of firearm ownership, including τ and η, whose role was deemed negligible in the calibration. However, to demonstrate that these variables do not influence our results, we performed an additional analysis without them (Table S5).

The model's output was evaluated relative to the GPSS estimates of firearm ownership (Figure 2). For each state, the model output for the month of October was subtracted from the fraction of firearm owners in the same month, and the difference was squared. Then, the sum of squared errors (SSE) and the mean of squared errors (MSE) were computed. On a national level, the SSE was 0.1436, and the MSE was 0.0072, suggesting that the model and survey responses are in agreement. The results for state-level computations are reported in Table S2.

Causal analysis using model predictions

To demonstrate the value of our model, we used its output in a causal analysis, exactly as done by Porfiri et al.[22] In their study, they showed the causal relationships within the fundamental triad of firearm prevalence, mass shootings, and media output on firearm control, using the information-theoretic notion of transfer entropy. Transfer entropy is a model-free approach for inference of causal relationships between pairs of dynamic

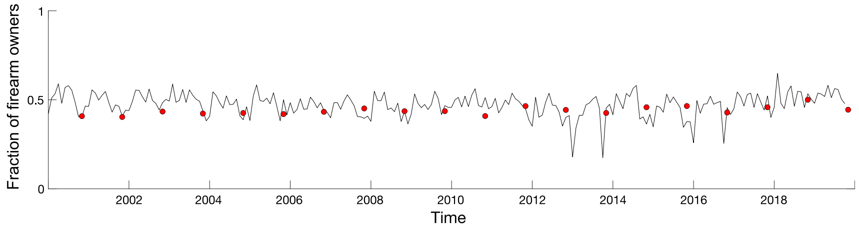

Figure 2. Predicted fraction of firearm owners in the United States

For a Figure360 author presentation of this figure, see https://doi.org/10.1016/j.patter.2022.100546. Figure360

The plot illustrates the model's output between January 2000 and December 2019 for the entire country. It is overlaid with GPSS survey annual results, represented by red circles.

systems. First introduced in 2000 by Schreiber, transfer entropy quantifies the extent to which uncertainty in the prediction of a future state of a system is reduced, given additional knowledge about its present state and the present state of another system.[43,44] It also supports the inference of causal links in the presence of nonlinear interactions and multiple time delays,[45,46] and it has been successfully implemented in a wide range of applications, including neuroscience,[45] economics,[47] animal behavior,[48] and human behavior.[49]

Following the procedures carried out by Porfiri et al.,[22] we aimed to uncover causal relationships in the triad of background checks, mass shootings, and media output and substituted background checks with our model's estimate of firearm ownership. Toward a complete comparative analysis, we also examined three triads capturing the relationships between mass shootings and media output on regulations with one of three variables: background checks (as in Porfiri et al.[22]), background checks per capita, and fraction of suicides committed with firearms. Since our model produced a time series beginning in January 2000, and the time series for mass shootings, media output, and background checks considered by Porfiri et al.[22] ended in December 2017, only the months between January 2000 and December 2017 were considered in the analysis. Therefore, each time series contained a total of 216 observations.

State-level background checks and background checks per capita showed strong seasonality, and suicides with firearms and our model's output showed trends in most states (Figures S1–S4). An augmented Dickey-Fuller test was applied to ensure stationarity of the processed time series (Table S3). Thus, as done previously by Porfiri et al.,[22] the time series of the four firearm variables (background checks, background checks per capita, fraction of suicides committed with firearms, and our model's output) were seasonally adjusted using the time series regression with ARIMA noise, missing values and outliers/signal extraction in ARIMA time series (TRAMO/SEATS) algorithm[50] and then linearly detrended by subtraction of their linear fit .

Next, we computed transfer entropy for each pair of variables under consideration, by conditioning on the other variable in the triad. Figure 3 displays the time series of processed background checks, background checks per capita, fraction of suicides with firearms, as well as the time series for mass shootings and media output on firearm control that were used in this analysis. The mass shootings we considered are listed in Table S4. Transfer entropy was calculated at the state level using each state's respective time series for background checks, background checks per capita, suicides with firearms, and firearm ownership. For nation-level analyses, the time series were aggregated across the 48 states (excluding Alaska and Hawaii) for each month. Finally, we performed a permutation test for each link under examination to assess whether transfer entropy values were different from chance.[51,52] All procedures related to transfer entropy and permutation tests were replicated from Porfiri et al.[22]

Results for causal analyses on a national level are summarized in Table 2 and Figure 4. Similar to the findings by Porfiri et al.,[22] we identified a causal link between media output and background checks ($p = 0.0317$) but not for the other variable pairs in the triad. When replacing background checks with the measure of background checks per capita or fractions of suicides with firearms, this causal link became non-significant ($p = 0.1546$ and $p = 0.5566$, respectively). When considering the triad with our model output, influence from media output to firearm ownership was marginally significant ($p = 0.0768$), and two other causal relationships emerged in the triad: the influence of firearm ownership on mass shootings ($p = 0.0136$) and on media output ($p = 0.0031$).

State-level transfer entropy is shown in Figure 5. Inspection of the significant conditional transfer entropies on a state level provided insights regarding the states where directional interactions were most predominant (Figure 5). Specifically, conditional transfer entropy from firearm ownership to mass shootings seemed to concentrate in states located in the West and Southwest regions as well as in the Midwest (Figure 5A). In contrast, conditional transfer entropy from firearm ownership to media output appeared to be strongest in the Southeast and Midwest (Figure 5B). Conditional transfer entropy from media output to firearm ownership was particularly eminent in the Southeast (Figure 5C).

To verify that causal links surfaced because of spatial interactions in our model, we generated a nation-level time series for the null model without W, whose parameters are reported in Table S1. In the absence of spatial interactions, this time series linearly combines the background checks per capita and suicides with firearms of each state. We computed transfer entropy for each pair of variables in a triad of the null model's output, mass shootings, and media output (Table S6). The analysis yielded no causal links, confirming that spatial interactions are crucial for detection of causal links.

DISCUSSION

Grounded in spatial econometrics, we created a spatiotemporal model that estimates state-level firearm ownership. The model specifies the interactions between states based on their geographical proximity and relative population size. Calibration of the model parameters provided some insight regarding firearm ownership processes that take place in the United States. With respect to the independent variables, background

CellPress
OPEN ACCESS

Table 1. Estimates for the model parameters

Parameter	Units	Estimate	*t*-Statistic	
ρ	[1]	0.1630	1.9342	$\circ$
τ	[1]	0.0034	0.1048	
η	[1]	−0.0493	−0.2546	
$\phi^{(1,H)}$	[\background checks]	18.1596	2.4757	$*$
$\phi^{(1,L)}$	[\background checks]	36.5954	7.9781	$*$
$\phi^{(2,H)}$	[1]	0.5285	6.3517	$*$
$\phi^{(2,L)}$	[1]	0.2741	4.7466	$*$
$\psi^{(1)}$	[\background checks]	−70.2457	−4.3252	$*$
$\psi^{(2)}$	[1]	1.5989	4.6192	$*$
α^H	[1]	−0.6225	−8.5281	$*$
α^L	[1]	−0.5080	−8.2259	$*$
γ	[1]	0.0104	7.6297	$*$
σ^2	[1]	0.0310	–	

The *t*-statistic and *p* value associated with each estimate indicate whether the parameter value is significantly different than zero. $\circ$ indicates a trend with $0.05 < p < 0.1$, and $*$ indicates a significance with $p < 0.05$.

checks per capita and fraction of suicides with firearms had strong predictive value in the model. Background checks had a direct influence on the prediction of firearm ownership. This effect was extended to spatial interactions between states, where the prediction of firearm ownership in a state was improved by knowledge of the number of background checks per capita in other states. This finding suggests that firearms cross state borders, an aspect that may be considered by legislators formulating new policies.

With respect to the fraction of suicides committed with firearms, it appears that this variable had direct and indirect effects through interactions between states. This finding is in line with past studies that examined patterns of suicides in the United States and found a spatial autocorrelation.[53,54] Nonetheless, spatial autocorrelation of suicides may be confounded by other factors that influence firearm ownership, such as religion, income, or education,[54–56] and warrant further investigation.

Inspection of our model also provides insight regarding autoregressive features of firearm ownership so that its measurement in one point in space or time is related to firearm ownership in another point in space or time. There appears to be contemporaneous spatial autoregression, where firearm ownership gradually changes over geographical locations. At the same time, temporal autoregression (that is, memory) was not registered, whether within states or across states. This finding suggests that firearm ownership is independent of its own history. However, it is tenable that memory effects were overshadowed by the time trend we introduced into the model. The coefficient for the linear time trend (γ) was non-zero, indicating that the interplay between variables is unique for every year. Therefore, the dummy variables we introduced for each year may have captured, in part, some of the memory effects in our model.

We used the model to infer firearm ownership in each state every month between January 2000 and December 2019. Then, we challenged our model's output in an information-theoretic framework. Specifically, we revisited one of our recent studies where we used transfer entropy to uncover causal relationships between firearm prevalence, mass shootings, and media output on firearm regulations.[22] Transfer entropy is a powerful and versatile tool for inference of causal relationships between pairs of dynamic systems from their time series,[43,57] quantifying the extent to which the predicted firearm ownership causally interacts with mass shootings and media output. Our group has previously implemented transfer entropy in the context of public health and policy, related and unrelated to firearm control.[22,34–36,58] In our previous examination of the mass shootings/media output/background checks triad, we found robust entropy transfers from media output to background checks, suggesting that media coverage is causally associated with the public's response to forthcoming stringent firearm control, in part driving firearm acquisition.[22,58] To conduct a complete comparison of our model against theirs, we examined four triads.

First we tested the interactions between mass shootings, media output, and background checks. Even though we used a shorter time series in the analysis (beginning in January 2000 instead of January 1999), we were able to replicate the inference of a causal link from media output to background checks. Next, we performed the exact same analysis, substituting the background check time series with that of background checks per capita and fraction of suicides with firearms. In both analyses, no causal relationships were identified. A few concerns arise from this finding that may warrant further research. First, the absence of significant interactions in the triad when the widely accepted measure of suicides is used brings to question its validity. So far, research using this metric was limited to correlational analyses. It is tenable that the link between suicides with firearms and firearm ownership is mediated or moderated by another factor. In this case, suicides with firearms would likely provide some insight into firearm ownership but must not be used as the sole predictor of firearm ownership. Second, the loss of significance when standardizing background check data with respect to state population brings to question whether such standardization is needed in causal analyses and otherwise. If firearm owners indeed tend to accumulate firearms in their households (as suggested earlier), then standardization of firearm measures with respect to the entire state's population would not be representative of its population. It is possible that a more spatially granular analysis needs to be performed to answer this question.

In our final analysis, we investigated the triad with our model's prediction of firearm ownership. The analysis yielded results similar to the findings by Porfiri et al.,[22] with marginal loss of significance for transfer entropy from media output to firearm ownership. This interaction was particularly evident in the Northwest and Southeast regions, where states are more permissive with respect to firearm laws.[59,60] It is tenable that media coverage of looming regulations particularly affects residents of permissive states, where there is more room for firearm control and new restrictive policies are more likely to materialize.

By including our model's prediction, however, two causal relationships have emerged in this analysis. Transfer entropy from firearm ownership to mass shootings supports the long-standing notion that perpetrators can commit their acts (especially spontaneous ones driven by emotion) because they have access to firearms.[7,61] In fact, in 71% of mass shootings, the firearms

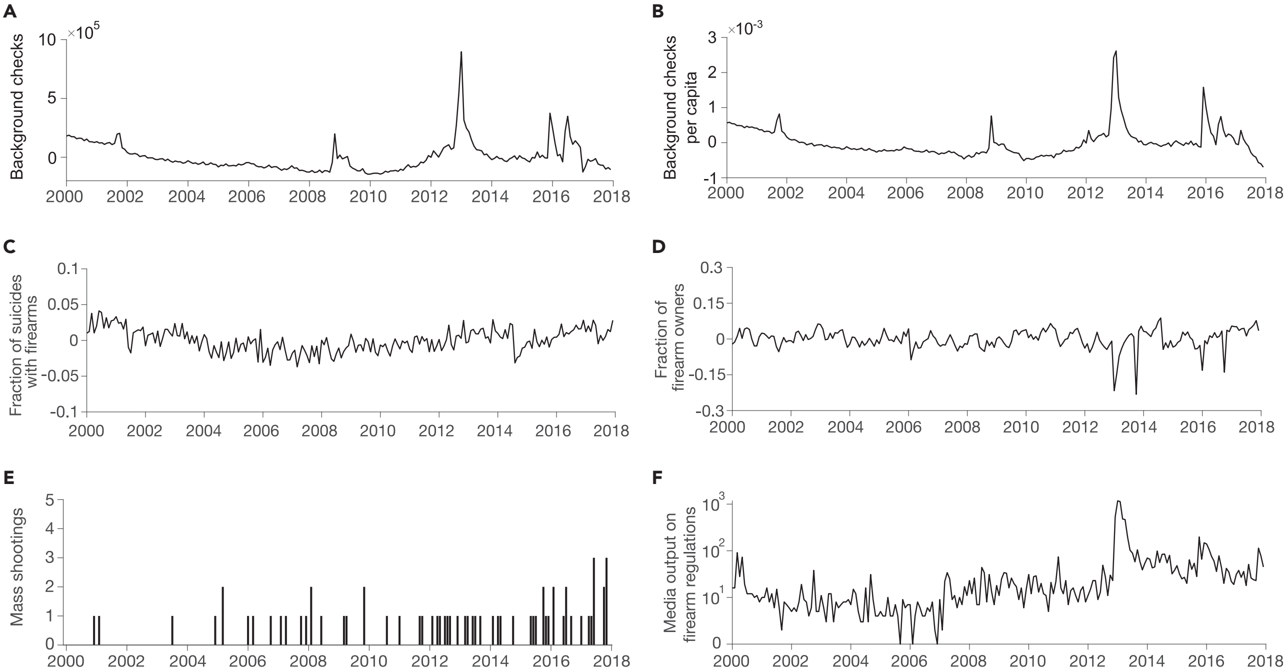

Figure 3. Processed time series for computation of transfer entropy

(A–F) Nationally aggregated time series between January 2000 and December 2017 for background checks (A), background checks per capita (B), fraction of suicides commited with firearms (C), fraction of firearm owners (D) were seasonally adjusted and detrended. The time series for mass shootings (E) was discretized, and the time series for media output on firearm regulations (F), presented on a logarithmic scale, remained unmodified in the analysis.

used were legally obtained and readily available to the perpetrators.[62] This causal relationship appears to concentrate in the West and Southwest, which is not unexpected considering that 37.5% of mass shootings took place in these parts of the country (Table S4). In addition, our analysis uncovered a causal link from firearm ownership to media output. Particularly in the Southeast and some Midwest states, there appears to be an association between firearm prevalence and public discourse on firearm regulations. It is possible that the way we measured media output as the integrated number of articles published in the New York Times and Washington Post introduced some bias. These news outlets likely report on firearm legislation in regions proximate to where they are circulated and are not representative of the entire nation. In future steps, we could consider extending media output to outlets that are more geographically and ideologically diverse to improve representation across the country.[58]

Although our work brings forward evidence in favor of using our model in firearm research, it has a number of limitations. First, we used GPSS survey responses as measurements of firearm ownership in the calibration. Although the GPSS probes for responses across the nation, the response rate is sometimes insufficient for estimating firearm ownership in less populated states.[6] Although we believe that the many data points considered in the maximum likelihood estimation mitigate this problem and point out that the spatial interaction components of the model extenuate such inaccurate values, one could use other means for calibration. For example, one might follow the path laid by Schell et al.[17] and use multi-level regression with post-stratification to establish a robust time series of firearm ownership for calibration. Alternatively, one might employ machine learning to improve the formulation of a spatial weight time series, but this approach remains under-explored.[63,64]

Second, we acknowledge that the model could benefit from inclusion of additional firearm ownership measures. For example, including the number of hunting licenses could improve the estimates of firearm ownership in states where outdoor recreational activities are practiced more commonly. However, introduction of additional variables into the model could undermined the power of maximum likelihood estimation because of the finite number of data points. In case one is interested in specific aspects of firearm ownership for policymaking purposes, one could substitute the independent variables of our model with alternative proxies. Nevertheless, we advise keeping the number of variables in the model to a minimum.

Finally, we would like to emphasize that our proposed model is specific to the United States and that its generalizability to other countries remains to be investigated. The unique federal structure of the United States is ideal for studying states' behaviors within the framework of spatial econometrics: states act as individual spatial units but share language, history, politics, and culture. In other settings, one could apply our methodology to cities or counties within a country, but too many dissimilarities may exist between international units. The relationships between firearm ubiquity and firearm violence may be unique to the United States. The United States experience 19.5, 5.8, and 5.2 times more homicides, suicides, and unintentional deaths, respectively, with firearms than other high-income countries.[65] In Switzerland, where firearm prevalence is among the highest in Europe (partly because of mandatory military conscription), firearm ownership translates to significantly lower rates of

Table 2. Conditional transfer entropy between the different variables on a national level

	Background checks	Mass shootings	Media output
Background checks	_	0.0159 (0.3481)	0.0057 (0.8206)
Mass shootings	0.0048 (0.8531)	_	0.0074 (0.7260)
Media output	0.0375 (0.0317) *	0.0133 (0.4428)	_
	Background checks per capita	Mass shootings	Media output
Background checks per capita	_	0.0146 (0.3930)	0.0082 (0.6748)
Mass shootings	0.0037 (0.9149)	_	0.0072 (0.7297)
Media output	0.0240 (0.1546)	0.0156 (0.3673)	_
	Fraction of suicides with firearms	Mass shootings	Media output
Fraction of suicides with firearms	_	0.0208 (0.2196)	0.0130 (0.4544)
Mass shootings	0.0129 (0.4581)	_	0.0149 (0.3846)
Media output	0.0106 (0.5566)	0.0160 (0.3459)	_
	Firearm ownership	Mass shootings	Media output
Firearm ownership	_	0.0464 (0.0136) *	0.0578 (0.0031) *
Mass shootings	0.0098 (0.5995)	_	0.0137 (0.4691)
Media output	0.0301 (0.0768) ∘	0.0230 (0.1818)	_

Rows represent sources, and columns represent targets. The numbers in parentheses denote the p value obtained from a permutation test. ∘ indicates a trend with $0.05 < p < 0.1$ and * a significance with $p < 0.05$.

harm, and most of it is self-inflicted rather than aimed toward others.[66] Such stark contrasts suggest that gun culture and other socioeconomic factors play a role in the realization of firearm violence in the United States.

Overall, we offer an avenue to generate knowledge of the American firearm ecosystem. Considering that the United States Constitution prohibits creation of a national registry of firearms, the scarcity of data on firearm prevalence remains an unsolved problem that hinders formulation of effective firearm policy. The absence of highly resolved data also prevents quantitative research on the effects of firearm prevalence on firearm violence that goes beyond simple correlational analyses. Hence, we provide a multivariate econometric model to estimate state-level firearm ownership on a monthly resolution from data of two proxies collected by government agencies (background checks and suicides committed with a firearm). Unlike previous efforts to estimate firearm prevalence, our model accounts for interactions between states and incorporates spatially and temporally autoregressive processes. Calibration of our model parameters indicated that both proxies have predictive value in estimation of prevalence and that interactions between states cannot be neglected. Finally, we demonstrated the utility of the model in uncovering causal relationships in information-theoretic analyses. For the first time, we unveil a causal link between mass shootings and firearm prevalence so that the model can help identify potential drivers of mass violence. Similar analyses inform policymakers about potential determinants and consequences of firearm ownership in every state, promoting design of effective legislation.

EXPERIMENTAL PROCEDURES

Resource availability

Lead contact

Requests for further information can be directed to the lead contact, M.P., at mporfiri@nyu.edu.

Materials availability

This study did not generate any materials.

Data and code availability

All data and codes needed to evaluate the conclusions in the paper are available on Github (https://doi.org/10.5281/zenodo.6582618).

Data collection

State-level data were collected for three variables for the years 2000–2019: fraction of firearm owners, background checks per capita, and fraction of suicides that were committed with a firearm. Data on background checks, mass shootings, and media output on firearm control on a monthly resolution were obtained from the Github repository compiled by Porfiri et al.[22] Data on firearm ownership and background checks were missing for Alaska and Hawaii, respectively. Along with the District of Columbia and the five United States territories, these states were excluded from the analysis.

Firearm ownership

Respondent-level data on firearm ownership were collected from the GPSS: Crime surveys.[18] These data were collected by Gallup staff every October by phone, and subjects were asked two questions: "Do you have a gun in your home?" and "Do you have a gun anywhere else on your property, such as in your garage, barn, shed, or in your car or truck?". Subjects had four possible answers: "Yes," "No," "I do not know," or refuse to respond. In total, 18,274 responses were recorded for each of the questions in the time period of 2000–2019. For the purpose of quantifying firearm ownership, we considered subjects who responded positively to at least one of those two questions as firearm owners. We took the number of firearm owners and divided it by the number of all subjects in the same state and year to yield the fraction of firearm owners. Firearm ownership data were not available for Alaska. A total of 931 measurements were collected for firearm ownership.

Background checks per capita

Data on background checks were collected on a monthly resolution from the FBI's NICS.[19] The NICS was established in November 1998, following legislation of the Brady Handgun Violence Prevention Act, mandating authorized firearm vendors to submit a background check request to determine whether a prospective buyer is eligible to purchase a firearm. Therefore, the number of background checks reports in the system also include also non-purchase counts. To better approximate the number of acquired firearms, we included only counts of permits for "handgun," "long gun," "other" firearms that are

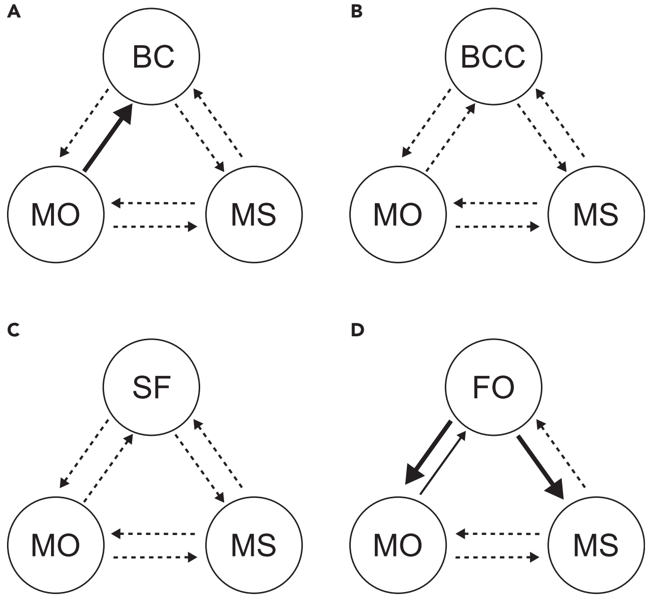

Figure 4. Directional interactions in four triads, quantified using transfer entropy

(A–F) Causal analysis results for (A) interactions between background checks (BCs), media output (MO) on firearm regulations, and mass shootings (MS); (B) interactions between background checks per capita (BCC), MO, and MS; (C) interactions between the fraction of suicides committed with firearms (SF), MO, and MS; (D) interactions between our model's firearm ownership (FO), MO, and MS. Dashed arrows reflect non-significant transfer entropy ($0.1 < p$), thin solid arrow indicate a trend ($0.05 < p < 0.1$), and bold solid arrows represent significant transfer entropy ($p < 0.05$).

not handguns or long guns (such as rifles or shotguns), and "multiple" types of firearms. Background checks administered for permit re-checks, pawns, redemptions, and rentals were excluded because they are not associated with newly acquired firearms. The number of background checks was standardized with respect to the state's population size by dividing each entry by the number of its inhabitants in the same year, obtained from the United States Census Bureau.[67,68] Background check data were not available for the state of Hawaii, so a total of 11,172 measurements were collected for background checks per capita.

Fraction of suicides with firearms

Data on suicides and their underlying causes were collected from the CDC's Wonder database.[4] Wonder's national mortality and population database is managed by the National Center for Health Statistics based on death certificates for United States residents. The database allows us to filter for death rates based on place of residence (state and county when available), age group, race, sex, and cause of death, distinguishing 113 selected causes of death for adults. We collected the total number of suicides by specifying "intentional self-harm" as the cause of death and grouped the results by state, year, and month. Then, we collected the number of suicides committed by "handgun discharge," "rifle, shotgun, and larger firearm discharge," or "other and unspecified firearm discharge," grouped by state, year, and month. The number of suicides committed with a firearm was divided by the total number of suicides to obtain the fraction of suicides with firearms. Overall, 11,400 measurements were collected for this variable.

Maximum likelihood estimation of the model

The econometric model was calibrated using maximum likelihood estimation, following LeSage and Pace.[42] In the estimation, we were limited by the resolution of firearm ownership, which is only available for the month of October. Therefore, we redefined the vectors of Equation (4) as

$$\mathbf{Y} = \begin{bmatrix} Y_{22} \\ Y_{34} \\ \vdots \\ Y_{12(T-1)+10} \end{bmatrix} \quad \mathbf{Y}_{\mathcal{L}} = \begin{bmatrix} Y_{10} \\ Y_{22} \\ \vdots \\ Y_{12T-2} \end{bmatrix} \quad \mathbf{WY}_{\mathcal{L}} = \begin{bmatrix} Y_{10} \\ Y_{22} \\ \vdots \\ Y_{12T-2} \end{bmatrix}$$

$$\mathbf{X}^{(j,R)} = \begin{bmatrix} X_{21}^{(j,R)} \\ X_{33}^{(j,R)} \\ \vdots \\ X_{12(T-1)+9}^{(j,R)} \end{bmatrix} \quad \mathbf{WX}^{(j,R)} = \begin{bmatrix} W_{21}X_{21}^{(j,R)} \\ W_{33}X_{33}^{(j,R)} \\ \vdots \\ W_{12(T-1)+9}X_{12(T-1)+9}^{(j,R)} \end{bmatrix}$$

where $j = 1,2$ represents background checks per capita and fraction of suicides committed with firearms, $R = H,L$ reflects states with high or low response rates, $T = 20$ is the number of years for which data is considered, and the subscript $\mathcal{L}$ denotes a time lag of 1 year. Then, the model to be estimated remains as

$$\mathbf{Y} = \mathcal{W}\mathbf{Y} + \delta\mathbf{Z} + \boldsymbol{\varepsilon} \qquad \text{(Equation 6)}$$

where

$$\mathbf{Z} = \left[\mathbf{Y}_{\mathcal{L}}\ \mathbf{WY}_{\mathcal{L}}\ \mathbf{X}^{(1,H)}\ \mathbf{X}^{(1,L)}\ \mathbf{X}^{(2,H)}\ \mathbf{X}^{(2,L)}\ \mathbf{WX}^{(1)}\ \mathbf{WX}^{(2)}\ \mathbf{d}_{(T-1)n}\ \mathbf{i}_{(T-1)n}^{(H)}\ \mathbf{i}_{(T-1)n}^{(L)}\right], \qquad \text{(Equation 7)}$$

$$\mathcal{W} = \begin{bmatrix} W_{22} & 0 & \cdots & 0 \\ 0 & W_{34} & \cdots & 0 \\ \vdots & \vdots & \cdots & \vdots \\ 0 & 0 & \cdots & W_{12(T-1)+10} \end{bmatrix}, \qquad \text{(Equation 8)}$$

$$\delta = \left[\tau\ \eta\ \phi^{(1,H)}\ \phi^{(1,L)}\ \phi^{(2,H)}\ \phi^{(2,L)}\ \psi^{(1)}\ \psi^{(2)}\ \gamma\ \alpha^{(H)}\ \alpha^{(L)}\right]', \qquad \text{(Equation 9)}$$

$\mathbf{d}_{(T-1)n}$ is a $(T-1)n$-dimensional vector of dummy variables containing a unique integer for each year, $\mathbf{i}_{(T-1)n}$ is a $(T-1)n$-dimensional vector of ones, and $\boldsymbol{\varepsilon}$ is an independent Gaussian noise of zero mean and covariance matrix $\sigma^2 I_{(T-1)n}$, with $I_{(T-1)n}$ being the identify matrix of size $(T-1)n$. The log likelihood function takes the form

$$\ln L = -\frac{(T-1)n}{2}\ln \gamma\sigma^2 + \ln\left|I_{(T-1)n} - \rho\mathcal{W}\right| - \frac{(\mathbf{Y} - \rho\mathcal{W}\mathbf{Y} - \mathbf{Z}\delta)'(\mathbf{Y} - \rho\mathcal{W}\mathbf{Y} - \mathbf{Z}\delta)}{2\sigma^2} \qquad \text{(Equation 10)}$$

where $\rho \in \left(\min(\omega)^{-1}, \max(\omega)^{-1}\right)$ and ω is an $(T-1)n$-dimensional vector of the eigenvalues of $\mathcal{W}$. In the estimations, the log determinant was approximated using a Monte Carlo scheme.[69] Through this iterative approach, a unit normal vector was randomly selected to estimate the trace of $\mathcal{W}$ so that the average of many estimated traces statistically approximated the true trace.[42,69] A Student's t-test was applied for each parameter estimate, indicating whether the parameter value was significantly different from zero.

Data pre-processing

In preparation for transfer entropy analysis, data were preprocessed in three successive steps: time series were seasonally adjusted, detrended, and transcribed to symbols.

Seasonal adjustment and detrending

Time series for each variable exhibited seasonality and lacked stationarity in many states (Table S3). Using them in their raw form in the information-theoretic framework would give rise to incorrect inference of interactions. To address this issue, we first seasonally adjusted the data using the TRAMO/SEATS method[50] on EViews (version 11, IHS Markit, London, UK). Assuming an autoregressive integrated moving average (ARIMA) model, TRAMO decomposes time series into long-term trend, a trend cycle, a seasonal component, and an irregular component. SEATS uses the ARIMA-based methodology to estimate unobserved components and reconstruct time series that are

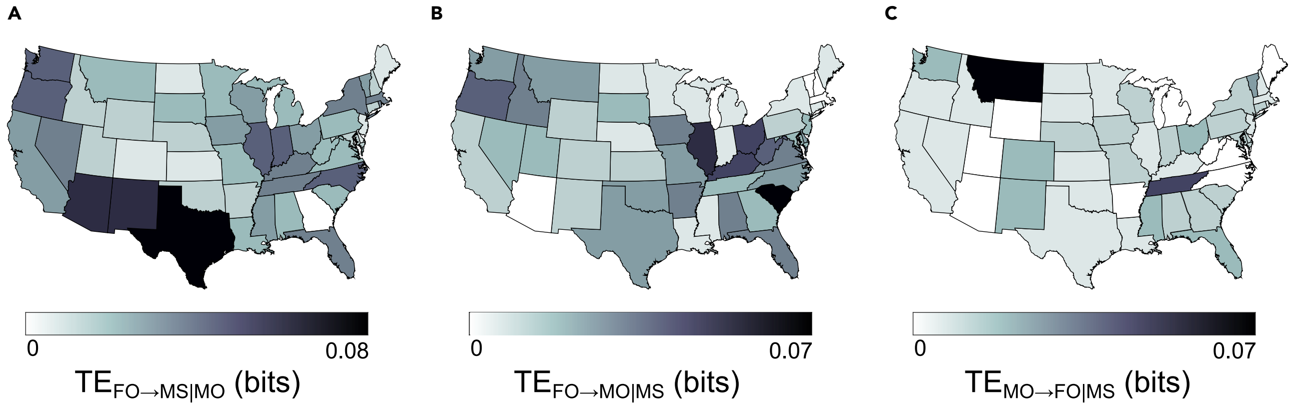

Figure 5. Causal analysis on a state level

(A–C) State-level conditional transfer entropy (A) from FO to MS, conditioned on MO; (B) from FO to MO, conditioned on MS; and (C) from MO to FO, conditioned on MS.

adjusted for trends and seasonal effects. For each state, the time series of each variable between January 2000 and December 2017 was taken at a time, decomposed, and seasonally adjusted. Then, it was detrended on MATLAB (MATLAB and Statistics Toolbox Release 2020a, MathWorks, Natick, MA, USA) by subtracting the linear fit of the time series, obtained with the "fitlm" function. Following this procedure, the augmented Dickey-Fuller test was used to ensure the stationarity of the processed time series.

Time series symbolization

To better capture the effect of variable changes during interactions, we pursued a symbolic approach.[57,70] For each variable, we created a new time series consisting of symbols that reflect changes between two successive measurements.[57,70] Specifically, for the variables background checks, background checks per capita, fraction of suicides committed with firearms, firearm ownership, and media output on firearm regulations, a value of 1 was assigned to time step t when the measurement at time step $t+1$ was greater than the one obtained at time step t. Otherwise, value of 0 was assigned. For mass shootings, a value of 1 was assigned when one or more mass shootings occurred in time step t, and a value of 0 was assigned when no mass shooting had occurred in that time step. Therefore, the symbolized time series at a given time step t indicated whether there was an increase or a decrease in the respective variables between t and $t+1$ and whether amass shooting took place at t. This scheme was applied consistently with the codes from Porfiri et al.,[22] as described in the associated Github readme file.

Conditional transfer entropy for causal analysis

Next, we computed transfer entropy for each pair of variables under consideration. The construct of transfer entropy is based on Shannon's notion of information as a measure of uncertainty.[71] For a discrete random variable X, Shannon's entropy takes the following form:

$$H(X) = -\sum_{x \in X} p(x) \log p(x), \quad \text{(Equation 11)}$$

where $p(x)$ is the probability that the random variable X takes value x, and Ω is the sample space of all possible outcomes of X. By specifying the logarithm with base 2, $H(X)$ is naturally given in bits. From a mathematical point of view, $H(X)$ can be viewed as the expectation of $-\log p(X)$. Therefore, we can define the joint and conditional entropies of two random variables X and Y as

$$H(X, Y) = -\sum_{x \in X, y \in Y} p(x,y) \log p(x,y) \quad \text{(Equation 12)}$$

and

$$H(X|Y) = -\sum_{x \in X, y \in Y} p(x, y) \log p(x|y), \quad \text{(Equation 13)}$$

where y is a realization of Y. The joint entropy can be interpreted as the overall uncertainty of both X and Y, whereas the conditional entropy can be understood as the amount of uncertainty of variable X, knowing the realization of Y.

Given Equations (12) and (13), it is possible to test the independence of X and Y through their mutual information,

$$I(X;Y) = H(X) - H(X|Y), \quad \text{(Equation 14)}$$

where the quantity $I(X;Y)$ will be equal to zero if X and Y are independent. Mutual information can be further extended to account for the presence of a third variable Z by computing conditional mutual information as

$$I(X;Y|Z) = H(X|Z) - H(X|Y, Z). \quad \text{(Equation 15)}$$

In a causal framework, we work with stationary stochastic processes. Transfer entropy from a process Y (source) to a process X (target) is computed as the reduction in uncertainty of predicting the future of X from its present, given knowledge about the present of Y:

$$TE_{Y \to X} = I(X_{t+1}; Y_t|X_t) = H(X_{t+1}|X_t) - H(X_{t+1}|X_t, Y_t). \quad \text{(Equation 16)}$$

$TE_{Y \to X}$ is a non-negative quantity; if Y is independent from X and does not encode useful information to predict it, then $H(X_{t+1}|X_t, Y_t)$ will equal $H(X_{t+1}|X_t)$ and transfer entropy will be zero.

Transfer entropy computes the dyadic influence between two processes. However, when dealing with multiple variables, simultaneous influences may lead to the inference of spurious interactions between non-interacting variables.[44] For instance, in this paper we deal with three variables: firearm prevalence, mass shootings, and media output. Should mass shootings influence firearm prevalence and media output, we may detect concurrent changes in firearm prevalence and media output and infer that they are coupled when in reality they may not be. Therefore, it is crucial that Equation (16) is adapted to account for a third variable. In this manner, conditional transfer entropy from Y to X, conditioned upon variable Z, can be computed as

$$TE_{Y \to X|Z} = I(X_{t+1}; Y_t|X_t, Z_t) = H(X_{t+1}|X_t, Z_t) - H(X_{t+1}|X_t, Y_t, Z_t). \quad \text{(Equation 17)}$$

Conditional transfer entropy was computed for all possible pairs within a triad by estimating the probability mass functions from the frequencies of symbols and evaluating the corresponding conditional joint entropies.

Computations assumed a first-order Markov process with a unitary time step (note subscripts t and $t+1$). Such a formulation would suggest that changes in one time series would lead to changes in another time series within a single month. To confirm that the time series are Markovian and that a single month is a sufficiently small time step, we performed additional statistical tests (see section S5 and S6 in the Supplemental experimental procedures). One month's timescale seems reasonable considering the

variables under inspection. Individuals will seek to purchase firearms in the month after the occurrence of a mass shooting or the breaking news of upcoming firearm regulations. Similarly, media output on firearm control will increase in the month after mass shooting events. Finally, because firearm prevalence has been repeatedly correlated with mass shootings in the United States, we would anticipate a causal link from the former to the latter within a month's time frame. In contrast, causal links from background checks to media output on regulation are not intuitively presumed, and the influence of media output on mass shootings is not expected because the latter are sporadic, individually motivated events. Nonetheless, one might consider the possibility of delayed interactions between the variables by incorporating time lags into the time series of Y and Z. In Figures S5 and S6, we present a delay analysis of the links that were found to be causal, with lags varying from 0 to 11 months. The results confirm that a unitary time step sufficiently captures the causal dynamics.

The significance of any interaction was determined by comparison with a surrogate distribution.[44,51] For each pair of variables, a local permutation scheme was carried out to preserve the conditioning of joint distributions upon a third variable.[51] Specifically, in the computation of each combination of $TE_{Y \rightarrow X|Z}$ in Equation (17), the subset of two-dimensional realization (X_t, Z_t) was taken. Then, the times series of Y_t in the same subset was randomly shuffled. This procedure was repeated for all possible realizations of (X_t, Z_t), (0,0), (0,1), (1,0), and (1,1), so that the entire time series of Y_t was randomly shuffled. Then, transfer entropy was computed with the shuffled time series. We performed this procedure 50,000 times and obtained 50,000 values of transfer entropy from which we constructed a surrogate distribution. The surrogate distribution would represent transfer entropy from one time series to another by chance from pairs of time series that were not causally associated in reality. To ensure that the computed value of transfer entropy from the observed time series is greater than chance, we checked whether it was in the right tail of the surrogate distribution. If it had exceeded its 95th percentile, then transfer entropy was considered to be non-zero.

SUPPLEMENTAL INFORMATION

Supplemental information can be found online at https://doi.org/10.1016/j.patter.2022.100546.

ACKNOWLEDGMENTS

This study was supported by National Science Foundation award CMMI-1953135 and by RAND's National Collaborative on Gun Violence Research through a postdoctoral fellowship award. The views expressed in this article are the authors' and do not necessarily reflect the view of the National Collaborative on Gun Violence Research. R.B.-V.'s work was supported in part by a Mitsui USA Foundation scholarship. This study was also part of the collaborative activities carried out under the programs of the region of Murcia (Spain): Groups of Excellence of the region of Murcia, Fundación Séneca, Science and Technology Agency project 19884/GERM/15. M.R.M. would like to acknowledge support from Ministerio de Ciencia e Innovación under grant PID2019-107800GB-I00/AEI/10.13039/501100011033.

AUTHOR CONTRIBUTIONS

Conceptualization, R.B.-V. and M.P.; methodology, R.B.-V., M.R.M., and M.P.; software, R.B.-V., M.R.M., and M.P.; validation, R.B.-V., M.R.M., and M.P.; formal analysis, R.B.-V., M.R.M., and M.P.; investigation, R.B.-V., M.R.M., and M.P.; resources, M.P.; data curation, R.B.-V.; writing – original draft, R.B.-V.; writing – review & editing, R.B.-V., M.R.M., and M.P.; visualization, R.B.-V.; supervision, M.P.; project administration, M.P.; funding acquisition, R.B.-V., M.R.M., and M.P.

DECLARATION OF INTERESTS

The authors declare no competing interests.

INCLUSION AND DIVERSITY

One or more of the authors of this paper self-identifies as an underrepresented ethnic minority in science.

Received: January 30, 2022
Revised: May 4, 2022
Accepted: June 6, 2022
Published: June 29, 2022

REFERENCES

1. Fowler, K.A., Dahlberg, L.L., Haileyesus, T., and Annest, J.L. (2015). Firearm injuries in the United States. Prev. Med. *79*, 5–14. https://doi.org/10.1016/j.ypmed.2015.06.002.
2. Vella, M.A., Warshauer, A., Tortorello, G., Fernandez-Moure, J., Giacolone, J., Chen, B., Cabulong, A., Chreiman, K., Sims, C., Schwab, C.W., et al. (2020). Long-term functional, psychological, emotional, and social outcomes in survivors of firearm injuries. J. Am. Med. Assoc. *155*, 51–59. https://doi.org/10.1001/jamasurg.2019.4533.
3. Lee, J., Quraishi, S.A., Bhatnagar, S., Zafonte, R.D., and Masiakos, P.T. (2014). The economic cost of firearm-related injuries in the United States from 2006 to 2010. Surgery *155*, 894–898. https://doi.org/10.1016/j.surg.2014.02.011.
4. CDC WONDER (2021). Underlying Cause of Death, 1999-2019. https://wonder.cdc.gov/ucd-icd10.html.
5. Duggan, M. (2001). More guns, more crime. J. Polit. Econ. *109*, 1086–1114. https://doi.org/10.1086/322833.
6. Kleck, G. (2004). Measures of gun ownership levels for macro-level crime and violence research. J. Res. Crime Delinquen. *41*, 3–36. https://doi.org/10.1177/0022427803256229.
7. Reeping, P.M., Cerdá, M., Kalesan, B., Wiebe, D.J., Galea, S., and Branas, C.C. (2019). State gun laws, gun ownership, and mass shootings in the US: cross sectional time series. Br. Med. J. *364*, l542. https://doi.org/10.1136/bmj.l542.
8. Nagin, D.S. (2020). Firearm availability and fatal police shootings. Ann. Am. Acad. Polit. Soc. Sci. *687*, 49–57. https://doi.org/10.1177/0002716219896259.
9. Wallace, L.N. (2020). Gun violence: an introduction. Violence Gend. *7*, 1–2. https://doi.org/10.1089/vio.2020.29025.lnw.
10. Dimock, M., Doherty, C., and Christian, L. (2021). Why own a gun? Protection is now top reason. https://www.pewresearch.org/politics/2013/03/12/why-own-a-gun-protection-is-now-top-reason/.
11. Gramlich, J., and Schaeffer, K. (2021). 7 facts about guns in the U.S. https://www.pewresearch.org/fact-tank/2019/10/22/facts-about-guns-in-united-states/.
12. Murray, M. (2018). Poll: 58 percent say gun ownership increases safety. https://www.nbcnews.com/news/amp/ncna859231.
13. Nagin, D.S., Koper, C.S., and Lum, C. (2020). Policy recommendations for countering mass shootings in the United States. Criminol. Publ. Pol. *19*, 9–15. https://doi.org/10.1111/1745-9133.12484.
14. Giffords Law Center (2021). Registration. https://giffords.org/lawcenter/gun-laws/policy-areas/owner-responsibilities/registration/.
15. National Center for Chronic Disease Prevention and Health Promotion (2020). The behavioral risk factor surveillance system Archived. https://www.cdc.gov/brfss/about/archived.htm.
16. Pew Research Center (2020). American Trends Panel Datasets. https://www.pewresearch.org/american-trends-panel-datasets/.
17. Schell, T.L., Peterson, S., Vegetabile, B.G., Scherling, A., Smart, R., and Morral, A.R. (2020). State-Level Estimates of Household Firearm Ownership. https://doi.org/10.7249/TL354.
18. Gallup. (2021). How does the Gallup Poll Social Series work? Long-term U.S. trends on social, economic, and political topics. https://www.gallup.com/175307/gallup-poll-social-series-methodology.aspx.

19. Federal Bureau of Investogation (2021). NICS firearm checks: month/year by state. https://www.fbi.gov/file-repository/nics_firearm_checks_-_month_year_by_state.pdf/view.

20. Ludwig, J., and Cook, P.J. (2000). Homicide and suicide rates associated with implementation of the Brady handgun violence prevention act. J. Am. Med. Assoc. *284*, 585. https://doi.org/10.1001/jama.284.5.585.

21. Wallace, L.N. (2015). Responding to violence with guns: mass shootings and gun acquisition. Soc. Sci. J. *52*, 156–167. https://doi.org/10.1016/j.soscij.2015.03.002.

22. Porfiri, M., Sattanapalle, R.R., Nakayama, S., Macinko, J., and Sipahi, R. (2019). Media coverage and firearm acquisition in the aftermath of a mass shooting. Nat. Human Behav. *3*, 913–921. https://doi.org/10.1038/s41562-019-0636-0.

23. Miller, M., Azrael, D., and Hemenway, D. (2002). Firearm availability and unintentional firearm deaths, suicide, and homicide among 5–14 year olds. J. Trauma *52*, 267–274. https://doi.org/10.1097/00005373-200202000-00011.

24. Denning, D.G., Conwell, Y., King, D., and Cox, C. (2000). Method choice, intent, and gender in completed suicide. Suicide Life-Threatening Behav. *30*, 282–288. https://doi.org/10.1111/j.1943-278X.2000.tb00992.x.

25. Brown, R.P., Imura, M., and Osterman, L.L. (2014). Gun culture: mapping a peculiar preference for firearms in the commission of suicide. Basic Appl. Soc. Psychol. *36*, 164–175. https://doi.org/10.1080/01973533.2014.882259.

26. Kõlves, K., McDonough, M., Crompton, D., and De Leo, D. (2018). Choice of a suicide method: trends and characteristics. Psychiatr. Res. *260*, 67–74. https://doi.org/10.1016/j.psychres.2017.11.035.

27. McDowall, D. (1991). Firearm availability and homicide rates in Detroit, 1951–1986. Soc. Forces *69*, 1085–1101. https://doi.org/10.2307/2579303.

28. McDowall, D. (1986). Gun availability and robbery rates: a panel study of large U.S. Cities, 1974–1978. Law Pol. *8*, 135–148. https://doi.org/10.1111/j.1467-9930.1986.tb00374.x.

29. Moody, C.E., and Marvell, T.B. (2003). Pitfalls of using proxy variables in studies of guns and crime. SSRN. https://doi.org/10.2139/ssrn.473661.

30. Kleck, G., and Patterson, E.B. (1993). The impact of gun control and gun ownership levels on violence rates. J. Quant. Criminol. *9*, 249–287. https://doi.org/10.1007/BF01064462.

31. Cook, P.J. (1991). The technology of personal violence. Crime Justice *14*, 1–71. https://doi.org/10.1086/449183.

32. Podobnik, B., and Stanley, H.E. (2008). Detrended cross-correlation analysis: a new method for analyzing two nonstationary time series. Phys. Rev. Lett. *100*, 084102. https://doi.org/10.1103/PhysRevLett.100.084102.

33. Morrison, C.N., Kaufman, E.J., Humphreys, D.K., and Wiebe, D.J. (2020). Firearm homicide incidence, within-state firearm laws, and interstate firearm laws in US counties. Epidemiology *32*, 36–45. https://doi.org/10.1097/EDE.0000000000001262.

34. Grabow, C., Macinko, J., Silver, D., and Porfiri, M. (2016). Detecting causality in policy diffusion processes. Chaos: An Interdisciplinary Journal of Nonlinear Science *26*, 083113. https://doi.org/10.1063/1.4961067.

35. Anderson, R.P., Jimenez, G., Bae, J.Y., Silver, D., Macinko, J., and Porfiri, M. (2016). Understanding policy diffusion in the US: an information-theoretical approach to unveil connectivity structures in slowly evolving complex systems. SIAM J. Appl. Dyn. Syst. *15*, 1384–1409. https://doi.org/10.1137/15M1041584.

36. Porfiri, M., and Marín, M.R. (2018). Information flow in a model of policy diffusion: an analytical study. IEEE Trans. Netw. Sci. Engineering *5*, 42–54. https://doi.org/10.1109/TNSE.2017.2731212.

37. Anselin, L. (2010). Thirty years of spatial econometrics. Pap. Reg. Sci. *89*, 3–25. https://doi.org/10.1111/j.1435-5957.2010.00279.x.

38. Anselin, L. (2013). In Spatial Econometrics: Methods and Models, *volume 4*.

39. Durbin, J. (1960). Estimation of parameters in time-series regression models. J. Roy. Stat. Soc. B *22*, 139–153. https://doi.org/10.1111/j.2517-6161.1960.tb00361.x.

40. Elhorst, J.P. (2010). Dynamic models in space and time. Geogr. Anal. *33*, 119–140. https://doi.org/10.1111/j.1538-4632.2001.tb00440.x.

41. Herrera Gómez, M., Mur Lacambra, J., and Ruiz Marín, M. (2012). Selecting the Most Adequate Spatial Weighting Matrix: A Study on Criteria (Munich Personal RePEc Archive).

42. LeSage, J., and Pace, R.K. (2009). Introduction to Spatial Econometrics (CRC Press).

43. Schreiber, T. (2000). Measuring information transfer. Phys. Rev. Lett. *85*, 461–464. https://doi.org/10.1103/PhysRevLett.85.461.

44. Bossomaier, T., Barnett, L., Harré, M., and Lizier, J.T. (2016). An Introduction to Transfer Entropy: Information Flow in Complex Systems (Springer International Publishing).

45. Vicente, R., Wibral, M., Lindner, M., and Pipa, G. (2011). Transfer entropy: a model-free measure of effective connectivity for the neurosciences. J. Comput. Neurosci. *30*, 45–67. https://doi.org/10.1007/s10827-010-0262-3.

46. Ito, S., Hansen, M.E., Heiland, R., Lumsdaine, A., Litke, A.M., and Beggs, J.M. (2011). Extending transfer entropy improves identification of effective connectivity in a spiking cortical network model. PLoS One *6*, e27431. https://doi.org/10.1371/journal.pone.0027431.

47. Papana, A., Kyrtsou, C., Kugiumtzis, D., and Diks, C. (2016). Detecting causality in non-stationary time series using partial symbolic transfer entropy: evidence in financial data. Comput. Econ. *47*, 341–365. https://doi.org/10.1007/s10614-015-9491-x.

48. Porfiri, M. (2018). Inferring causal relationships in zebrafish-robot interactions through transfer entropy: a small lure to catch a big fish. Animal Behav. Cognition *5*, 341–367. https://doi.org/10.26451/abc.05.04.03.2018.

49. Ventura, R.B., Richmond, S., Nadini, M., Nakayama, S., and Porfiri, M. (2021). Does winning or losing change players' engagement in competitive games? Experiments in virtual reality. IEEE Transactions on Games *13*, 23–34. https://doi.org/10.1109/TG.2019.2928795.

50. Maravall, A. (2006). An application of the TRAMO-SEATS automatic procedure; direct versus indirect adjustment. Comput. Stat. Data Anal. *50*, 2167–2190. https://doi.org/10.1016/j.csda.2005.07.006.

51. Runge, J. (2018). Causal network reconstruction from time series: from theoretical assumptions to practical estimation. Chaos: An Interdisciplinary Journal of Nonlinear Science *28*, 075310. https://doi.org/10.1063/1.5025050.

52. Chávez, M., Martinerie, J., and Le Van Quyen, M. (2003). Statistical assessment of nonlinear causality: application to epileptic EEG signals. J. Neurosci. Methods *124*, 113–128. https://doi.org/10.1016/S0165-0270(02)00367-9.

53. Zimmerman, S.L. (1987). States' public welfare expenditures as predictors of state suicide rates. Suicide Life-Threatening Behav. *17*, 271–287. https://doi.org/10.1111/j.1943-278X.1987.tb00068.x.

54. Wasserma, I.M., and Stack, S. (1995). Geographic spatial autocorrelation and United States suicide patterns. Arch. Suicide Res. *1*, 121–129. https://doi.org/10.1080/13811119508258980.

55. Chang, S.S., Sterne, J.A., Wheeler, B.W., Lu, T.H., Lin, J.J., and Gunnell, D. (2011). Geography of suicide in Taiwan: spatial patterning and socioeconomic correlates. Health Place *17*, 641–650. https://doi.org/10.1016/j.healthplace.2011.01.003.

56. Iyanda, A.E., Chima-Adaralegbe, N., Adeleke, R., and Lu, Y. (2021). Covariation of suicide and HIV in 186 countries: a spatial autoregressive and multiscale geographically weighted regression analyses. J. Publ. Health *30*, 1545–1555. https://doi.org/10.1007/s10389-020-01436-3.

57. Staniek, M., and Lehnertz, K. (2008). Symbolic transfer entropy. Phys. Rev. Lett. *100*, 158101. https://doi.org/10.1103/PhysRevLett.100.158101.

58. Porfiri, M., Barak-Ventura, R., and Marín, M.R. (2020). Self-protection versus fear of stricter firearm regulations: examining the drivers of firearm acquisitions in the aftermath of a mass shooting. Patterns *1*, 100082. https://doi.org/10.1016/j.patter.2020.100082.

59. Depetris-Chauvin, E. (2015). Fear of Obama: an empirical study of the demand for guns and the US 2008 presidential election. J. Publ. Econ. *130*, 66–79. https://doi.org/10.1016/j.jpubeco.2015.04.008.

60. Thompson, M. (2022). Why gun sales often rise after mass shootings. https://www.cnbc.com/id/100321785.

61. Hong, J.S., Cho, H., and Lee, A.S. (2010). Revisiting the Virginia Tech shootings: an ecological systems analysis. J. Loss Trauma *15*, 561–575. https://doi.org/10.1080/15325024.2010.519285.

62. Luca, M., Malhotra, D., and Poliquin, C. (2020). The impact of mass shootings on gun policy. J. Publ. Econ. *181*, 104083. https://doi.org/10.1016/j.jpubeco.2019.104083.

63. Cerulli, G. (2021). Improving econometric prediction by machine learning. Appl. Econ. Lett. *28*, 1419–1425. https://doi.org/10.1080/13504851.2020.1820939.

64. Kopczewska, K. (2021). Spatial machine learning: new opportunities for regional science. Ann. Reg. Sci. *68*, 713–755. https://doi.org/10.1007/s00168-021-01101-x.

65. Richardson, E.G., and Hemenway, D. (2011). Homicide, suicide, and unintentional firearm fatality: comparing the United States with other high-income countries, 2003. J. Trauma *70*, 238–243. https://doi.org/10.1097/TA.0b013e3181dbaddf.

66. Killias, M., and Markwalder, N. (2012). Firearms and homicide in Europe. In Handbook of European Homicide Research, pp. 261–272. https://doi.org/10.1007/978-1-4614-0466-8_16.

67. U.S. Census Bureau (2021). State Intercensal Tables: 2000-2010. https://www.census.gov/data/tables/time-series/demo/popest/intercensal-2000-2010-state.html.

68. U.S. Census Bureau (2021). State Population Totals: 2010-2019. https://www.census.gov/data/datasets/time-series/demo/popest/2010s-state-total.html.

69. Barry, R.P., and Kelley Pace, R. (1999). Monte Carlo estimates of the log determinant of large sparse matrices. Lin. Algebra Appl. *289*, 41–54. https://doi.org/10.1016/S0024-3795(97)10009-X.

70. Porfiri, M., and Ruiz Marín, M. (2017). Symbolic dynamics of animal interaction. J. Theor. Biol. *435*, 145–156. https://doi.org/10.1016/j.jtbi.2017.09.005.

71. Shannon, C.E. (1948). A mathematical theory of communication. Bell Labs Tech. J. *27*, 379–423. https://doi.org/10.1002/j.1538-7305.1948.tb01338.x.

Patterns, Volume *3*

Supplemental information

A spatiotemporal model of firearm ownership in the United States

Roni Barak-Ventura, Manuel Ruiz Marín, and Maurizio Porfiri

Supplemental procedures

S1. State-level time series

We present the raw time series for background checks (BC; Fig. S1), background checks per capita (BCC; Fig. S2), and fraction of suicides that were committed with firearms (SF; Fig. S3), in each state. We also include the prediction of our model for the fraction of firearm owners in each state, overlaid with the fraction of firearm owners reports by the Gallup Poll Social Series (GPSS) every October (FO; Fig. S4).

S2. Exploring different spatial weight matrices

Next, we summarize the results for calibration of our model with different spatial weight matrices (Table S1). Specifically, we considered spatial weight matrices that encapsulate the relationships between states based on their distance (D), area (A), population size (p), gross domestic product (GDP), and presence of a border (B). This endeavor was carried out with the underlying notion that states that are more proximate, vast, populated, economically productive, or share a border, may exert more influence on other states.
To facilitate the selection of a weight matrix for our analysis, we examined the autoregressive terms and residuals of each model. Inspection of the results revealed that the terms τ and η were not significantly different from zero for any of the models. The autoregressive term ρ emerged only for the models that contain D or B. Among those models, the lowest σ^2 was registered for the one with W containing $\frac{p_j}{D_{i,j}}$ in its elements. Thus, this spatial weight matrix was selected for our study.

S3. Evaluating the model output

Once the model output was obtained, the predicted FO in each state every October was compared to the corresponding fraction of firearm owners reported by the GPSS by computing the sum of squared errors (SSE) and the mean of squared errors (MSE). The smallest errors occurred in highly populated states such as New York, California, and Florida. In contrast, the largest errors appeared in the least populated states, such as South Dakota, Vermont, and Wyoming (Table S9).

S4. Pre-processing time series

State-level BC and BCC exhibited non-stationarity and strong seasonality in all states. Therefore, before computing transfer entropy between any pair of time series, it was imperative to remove those patterns in order to prevent erroneous inference of causality. We determined stationarity of raw time series for each variable under consideration (BC, BCC, SF, and FO) in each state using an Augmented Dickey-Fuller test (Table S9). The test revealed that both variables were majorly non-stationary: only 11 states exhibited stationarity with respect to BC, 12 showed stationarity with respect to BCC, and 14 with respect to fraction of SF. In order to treat all states equally, all time series were seasonally adjusted and detrended. The Augmented Dickey-Fuller test was applied following this procedure to confirm that the time series were now stationary at a significance level of 0.05.
The time series of mass shootings was adapted from data collected by Mother Jones.[1] The data consist of 64 mass shooting events from January 2000 to December 2017 (Table S4). The monthly time series of mass shootings in our analysis was binary (consisting of zeros and ones), where 0 denoted no mass shootings took place and 1 reflected at least one mass shooting.[2]

S5. Testing for time series Markovianity

Before proceeding to transfer entropy analysis, we confirmed that the time series in our analysis are Markovian. Within the framework of mutual information, we computed

$$I(X_t; X_{t-2}|X_{t-1}, X_{t-3}) = H(X_t|X_{t-1}, X_{t-3}) - H(X_t|X_{t-1}, X_{t-2}, X_{t-3}) \quad \text{(S1)}$$

for each time series. Should the time series be Markovian, the quantity in equation S1 would be equal to zero. To verify that the value was not significantly different from zero, we performed a permutation test where we shuffled the time series of X_{t-2} and computed conditional mutual information as in equation S1. We repeated this procedure 50,000 times to obtain 50,000 values of mutual information. This surrogate distribution represented the conditional mutual information that would result from chance. We tested whether the observed value was not in the right tail of the surrogate distribution, within its 95th percentile. For the time series of BC, BCC, SF, and FO, we measured mutual information of magnitudes 0.0230, 0.0207, 0.0036, and 0.0147 bits, respectively. Those measurements were not significantly different from zero ($p = 0.1629$, $p = 0.2085$, $p = 0.9044$, and $p = 0.3832$, respectively). For the time series of MS and MO, mutual information assumed values of 0.0193 and 0.0249, respectively. These values were statistically indistinguishable from zero ($p = 0.2875$ and $p = 0.1351$, respectively).

S6. Testing for contemporaneous effects

In addition to assessing memory within time series, we also confirmed that the selection of one month's time step between measurements of our time series is appropriate. If causal relationships were to exist on a time interval smaller than a month, then spurious links could be inferred through conditional transfer entropy.[3] To exclude the possibility of such interactions, we tested for contemporaneous effects in each pair of variables X and Y within a triad. Specifically, we computed mutual information between contemporaneous time series X and Y at time step t, conditioned on the their past and the past of Z

$$I(X_t; Y_t|X_{t-1}, Y_{t-1}, Z_{t-1}) = H(X_t|X_{t-1}, Y_{t-1}, Z_{t-1}) - H(X_t|Y_t, X_{t-1}, Y_{t-1}, Z_{t-1}). \quad \text{(S2)}$$

In the absence of contemporaneous effects, the quantity in equation S2 should be equal to zero. To test whether this quantity was not statistically different from zero, we performed a permutation test. We shuffled the entire time series of X and Y such that any potential association between them is disrupted, and computed conditional mutual information as in equation S2. We computed 50,000 values of mutual information and generated a surrogate distribution representing the conditional mutual information from chance. Values that exceeded the 95th percentile of the surrogate distribution would be deemed significantly different from zero.

Within the benchmark triad of BC, MS, and MO, 0.0176 bits were computed between BC and MS, 0.0307 between BC and MO, and 0.0136 between MS and MO. None of these values was significantly different from zero ($p = 0.8306$, $p = 0.4376$, and $p = 0.9156$, respectively). For the triad containing BCC instead of BC, mutual information amounted to 0.0105 bits between BCC and MS, 0.0185 between BCC and MO, and 0.0094 between MS and MO. Here too, mutual information values were not significantly different from zero ($p = 0.9607$, $p = 0.7858$, and $p = 0.9724$, respectively). In the triad involving SF, MS, and MO, mutual information was measured at 0.0373 bits, 0.0359 bits, and 0.0297 bits for the links between SF and MS, SF and MO, and MS and MO, respectively. None of these measurements was statistically different from zero ($p = 0.3030$, $p = 0.3152$, and $p = 0.4890$, respectively). Finally, for the triad containing our measurement of FO, mutual information was 0.0288 bits between FO and MS, 0.0030 between FO and MO, and 0.0061 between MS and MO. None of those values was significantly different from zero ($p = 0.5142$, $p = 0.9900$, and $p = 0.9900$, respectively).

S7. Conditional transfer entropy with time delays

To challenge our model's output, we revisited one of our previous studies where causal links between firearm prevalence, MS, and MO were quantified and investigated.[2] Specifically, we computed transfer entropy between pairs of national-level time series and found four causal links: MO$\rightarrow$ BC|MS, FO$\rightarrow$ MS|MO, FO$\rightarrow$ MO|MS, and MO$\rightarrow$ FO|MS. In addition, we explored the possibility of delayed interactions between the variables by computing conditional transfer entropy with delays,

$$\mathrm{TE}_{Y\to X|Z} = I(X_{t+1}; Y_{t-\delta_Y}|X_t, Z_{t-\delta_Z}) = H(X_{t+1}|X_t, Z_{t-\delta_Z}) - H(X_{t+1}|X_t, Y_{t-\delta_Y}, Z_{t-\delta_Z}). \quad \text{(S3)}$$

where δ_Y and δ_Z are the delays used for Y and Z, respectively. In this setting, one delay can be used for the source variable (Y) and a different delay can be considered for the variable conditioned upon (Z). For each pairwise interaction, we independently varied δ_Y and δ_Z from 0 to 11 and computed the corresponding delayed conditional transfer entropy to obtain 12$\times$12 values. For example, considering transfer entropy from mass shootings (Y) to background checks (X) conditioned on media output (Z), we computed 12$\times$12 values of transfer entropy for delays in mass shootings and media output.

In Fig. S5, we highlight the results of these analyses for the links we found to be causal (MO$\rightarrow$ BC|MS, FO$\rightarrow$ MS|MO, FO$\rightarrow$ MO|MS, and MO$\rightarrow$ FO|MS). In each plots with blue markers on the left, we show the mean of 12 transfer entropy values with fixed delays on the source variable ranging from 0 to 11. The vertical bars extending from the markers represent the standard deviation when the delay on the conditional variable varies from 0 to 11. Similarly, in each of the plots with red markers, we show the means of 12 transfer entropy values with fixed delays on the conditional variable. There, vertical bars represent the standard deviation when delays are applied on the source variable.

For MO$\rightarrow$ BC|MS, FO$\rightarrow$ MS|MO, and MO$\rightarrow$ FO|MS, the largest transfer entropy value was observed for a delay of 0. For FO$\rightarrow$ MO|MS, transfer entropy is maximized for delays of 0 and 2 months. We further investigated the nature of the 2-months delay by examining transfer entropy on a state-level. Specifically, we hypothesized that the media's association with firearm prevalence would differ between permissive and restrictive states. In permissive states, there is more room for firearm legislation and MO on firearm control would focus on legislation in those states, whereas firearm control would be only marginally discussed with a longer delay in restrictive states. Thus, following our cluster analysis in,[4] we identified California, Connecticut, Illinois, Maryland, Massachusetts, New Jersey, and New York as restrictive, and all other states as permissive. For each delay ranging from 0 to 11, we computed a mean value of transfer entropy for all restrictive states (Fig. S6a) and for all permissive states (Fig. S6b), weighted by each state's population size. We found that transfer entropy from FO to MO is maximized for permissive states without a delay.

S8. Conditional transfer entropy in the absence of τ and η

In our prediction of FO, we accounted for all calibrated coefficients to improve the model's prediction. However, one might suggest that including coefficients that are not significantly different from zero in the model would create an over-specified model. To confirm that the inclusion of such terms does not compromise our inference of causal relationship, we generated a time series of firearm ownership on a national level between January 2000 and December 2017 with our model, specifying $\tau = 0$ and $\eta = 0$. We used the model output in transfer entropy analysis within the triad of FO, MS, and MO (Table S5) and verified that the results are similar to the findings in the main manuscript (Table 2).

S9. Conditional transfer entropy in the absence of spatial interactions

Finally, we aimed to demonstrate that spatial regression in our model gave rise to causal links in transfer entropy analysis. To this end, we inferred firearm ownership in each state between January 2000 and October 2019, using the calibrated parameters of a null model with $W = 0$ (first row in Table S1). In the absence of spatial interactions between states, the time series we generated linearly combined the

background checks per capita and fraction of suicides with firearms within a state to compute its own firearm ownership. We used the model output in transfer entropy analysis within the triad of FO/MS/MO and found that none of the links is significantly different from chance (Table S6). This result indicates that spatial interactions are crucial for the detection of causal links.

References

1. Follman, M., Aronsen, G., Pan, D. (2021), US Mass Shootings, 1982–2021: Data From Mother Jones' Investigation. URL https://www.motherjones.com/politics/2012/12/mass-shootings-mother-jones-full-data/.
2. Porfiri, M., Sattanapalle, R.R., Nakayama, S., Macinko, J., Sipahi, R. (2019). Media coverage and firearm acquisition in the aftermath of a mass shooting. Nature Human Behaviour, 3, 913–921, URL http://dx.doi.org/10.1038/s41562-019-0636-0.
3. Runge, J. (2020). Discovering contemporaneous and lagged causal relations in autocorrelated nonlinear time series datasets. In Conference on Uncertainty in Artificial Intelligence (PMLR), pp. 1388–1397.
4. Porfiri, M., Barak-Ventura, R., Marín, M.R. (2020). Self-protection versus fear of stricter firearm regulations: examining the drivers of firearm acquisitions in the aftermath of a mass shooting. Patterns, 1, 100082, URL http://dx.doi.org/10.1016/j.patter.2020.100082.

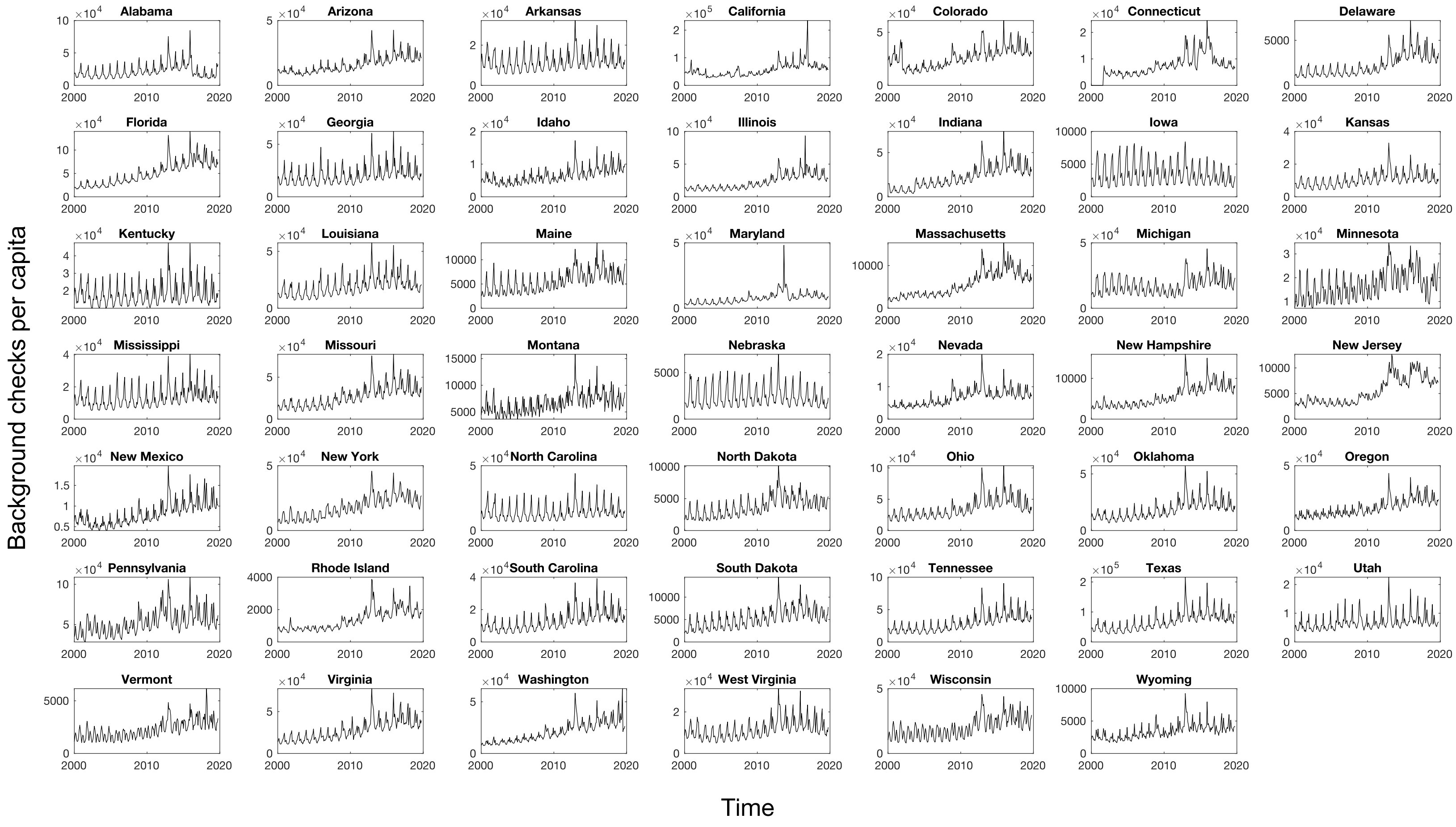

Figure S1: State-level background checks. Each panel displays the time series for monthly background checks in each U.S. state considered in the analysis. Alaska and Hawaii were excluded from the analysis due to missing data.

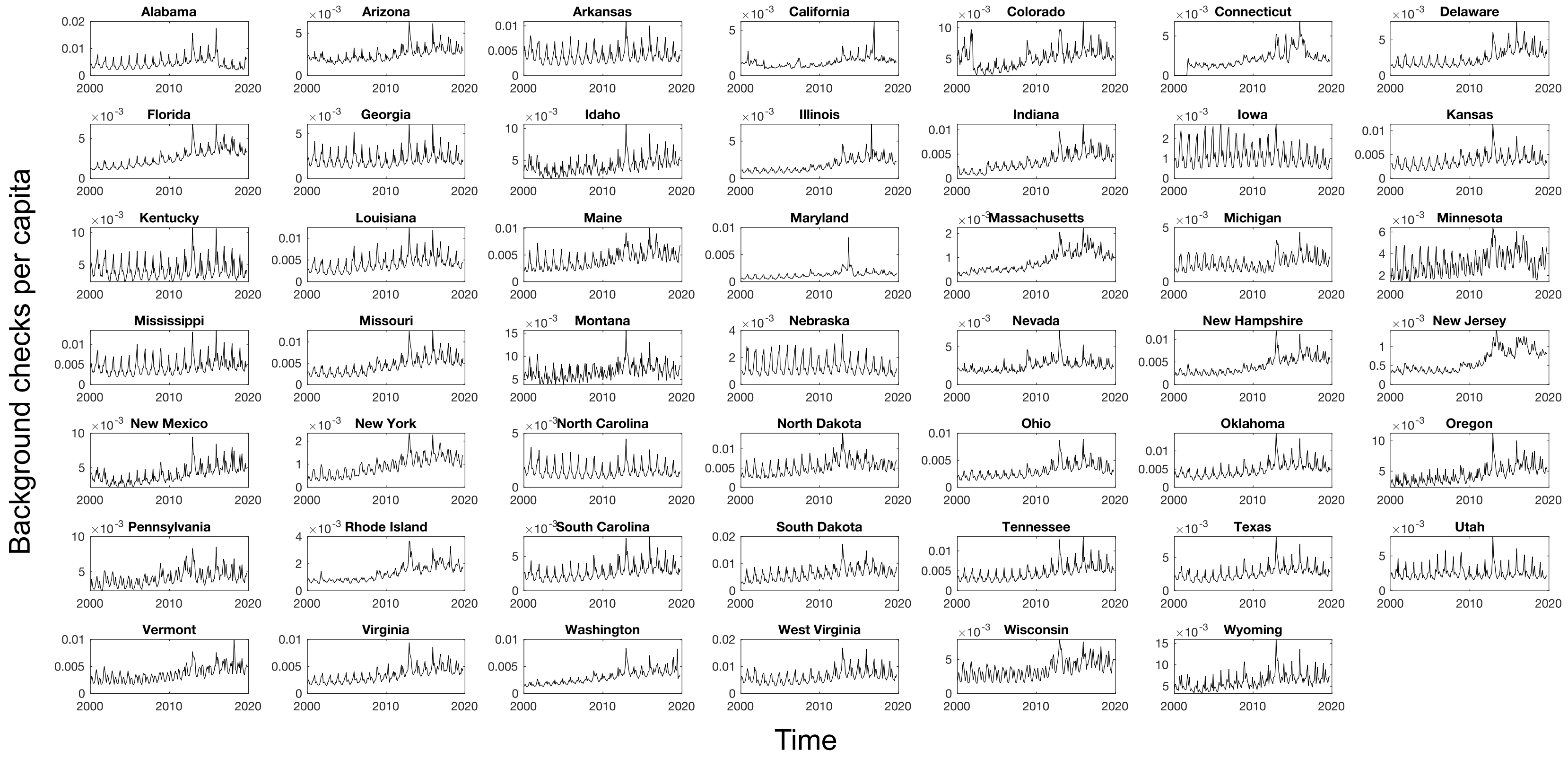

Figure S2: State-level background checks per capita. Each panel displays the time series for monthly background checks per capita in each U.S. state considered in the analysis. Alaska and Hawaii were excluded from the analysis due to missing data.

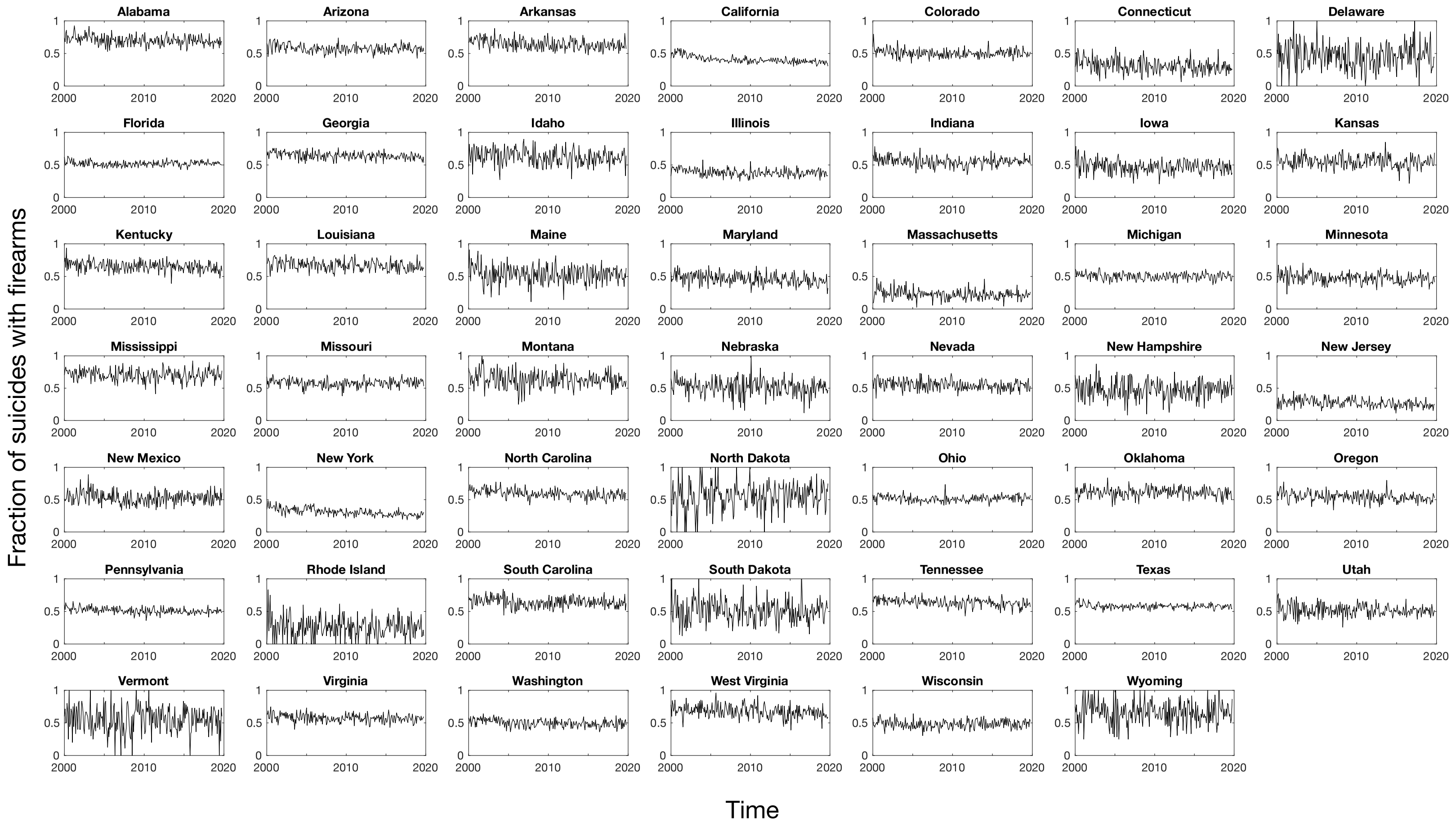

Figure S3: State-level suicides with firearms. Each panel displays the time series for the monthly fraction of suicides committed with firearms in each U.S. state considered in the analysis. Alaska and Hawaii were excluded from the analysis due to missing data.

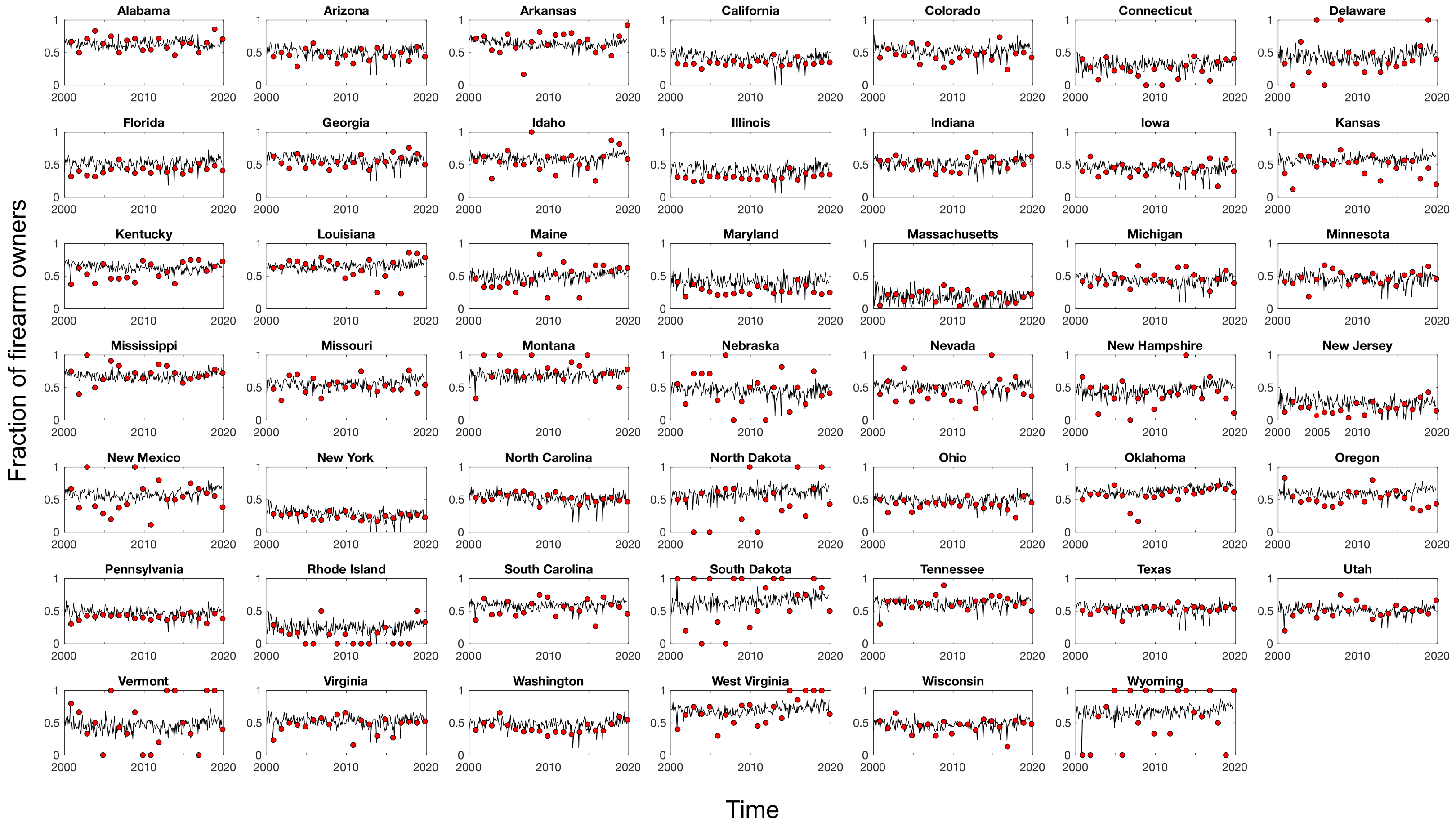

Figure S4: Predicted state-level firearm ownership. Each panel displays the model's output for monthly fraction of firearm owners in each U.S. state considered in the analysis. The red circles overlaying the time series reflect the fraction of firearm owners obtained from GPSS survey responses. Alaska and Hawaii were excluded from the analysis due to missing data.

$W_{i,j}$	ρ	τ	η	$\phi^{(1,\mathrm{H})}$	$\phi^{(1,\mathrm{L})}$	$\phi^{(2,\mathrm{H})}$	$\phi^{(2,\mathrm{L})}$	$\psi^{(1)}$	$\psi^{(2)}$	γ	$\alpha^{(1)}$	$\alpha^{(2)}$	σ^2
Null	N/A	0.0650<br>(2.0073)	N/A	14.9787<br>(1.9866)	33.1150<br>(7.0849)	0.6591<br>(7.7568)	0.4213<br>(7.5694)	N/A	N/A	0.0219<br>(0.5640)	0.1086<br>(3.4428)	0.0024<br>(2.1117)	0.0329
$\frac{1}{D_{i,j}}$	0.2120<br>(2.1290)	0.0111<br>(0.3378)	0.2037<br>(1.3012)	15.8757<br>(2.1169)	35.6671<br>(7.5810)	0.4956<br>(5.7229)	0.3035<br>(5.2556)	-49.1362<br>(-2.8931)	0.8756<br>(3.2641)	0.0058<br>(2.8418)	-0.3954<br>(-4.7000)	-0.3182<br>(-3.8803)	0.0314
A_j	-0.0190<br>(-0.0439)	0.0568<br>(1.8018)	0.0270<br>(0.0915)	17.8256<br>(2.3664)	34.2948<br>(7.4053)	0.6540<br>(7.8235)	0.4259<br>(7.6995)	-45.0485<br>(-2.2004)	-0.3368<br>(-1.0503)	0.3377<br>(0.5108)	0.4210<br>(0.6397)	0.0041<br>(2.0864)	0.0325
p_j	-0.0010<br>(-0.0038)	0.0555<br>(1.7301)	-0.6913<br>(-1.9739)	16.4106<br>(2.1853)	35.0488<br>(7.5007)	0.6302<br>(7.5426)	0.4233<br>(7.6791)	-67.0699<br>(-2.8422)	-0.1708<br>(-0.5053)	0.0081<br>(2.3780)	0.5318<br>(3.5903)	0.5981<br>(4.0656)	0.0323
$\frac{p_j}{A_j}$	0.0210<br>(1.4630)	0.0563<br>(1.7396)	-0.3133<br>(-1.9207)	16.8028<br>(2.2359)	34.8698<br>(7.4714)	0.6316<br>(7.4828)	0.4178<br>(7.5422)	-79.3382<br>(-3.4242)	0.0406<br>(0.1155)	0.0089<br>(3.4519)	0.2214<br>(1.4666)	0.2943<br>(1.9815)	0.0324
$\frac{p_j}{D_{i,j}}$	0.1600<br>(4.2194)	0.0034<br>(0.1064)	-0.0489<br>(-0.1738)	18.1607<br>(2.4782)	36.5966<br>(8.0076)	0.5285<br>(6.3866)	0.2742<br>(4.7925)	-70.2875<br>(-4.6749)	1.6014<br>(4.0111)	0.0104<br>(5.8702)	-0.6226<br>(-10.1421)	-0.5081<br>(-10.6513)	0.0310
$\frac{p_j}{D_{i,j}A_j}$	0.1760<br>(1.9553)	0.0155<br>(0.4762)	-0.0100<br>(-0.0714)	14.5327<br>(1.9608)	35.1491<br>(7.5903)	0.5176<br>(6.0526)	0.3183<br>(5.5524)	-38.0757<br>(-1.8346)	0.8851<br>(3.5212)	0.0068<br>(2.8018)	-0.2753<br>(-4.2243)	-0.2016<br>(-3.1926)	0.0313
GDP_j	-0.0010<br>(-0.0037)	0.0557<br>(1.7354)	-0.6909<br>(-1.9285)	16.3119<br>(2.1723)	35.0451<br>(7.4988)	0.6257<br>(7.4988)	0.4236<br>(7.6831)	-69.4314<br>(-2.9345)	-0.1163<br>(-0.3799)	0.4963<br>(3.1898)	0.5603<br>(3.6383)	0.0080<br>(2.3640)	0.0323
$\frac{GDP_j}{D_{i,j}}$	0.1330<br>(1.6031)	0.0011<br>(0.0342)	-0.1353<br>(-0.7012)	17.8700<br>(2.4393)	36.5014<br>(7.9754)	0.5354<br>(6.4673)	0.2741<br>(4.7887)	-69.9531<br>(-4.3995)	1.6713<br>(4.4609)	-0.6060<br>(-10.4091)	-0.4899<br>(-10.8460)	0.0115<br>(7.0143)	0.0309
$\frac{GDP_j}{A_{i,j}}$	0.0170<br>(1.4089)	0.0575<br>(1.7812)	-0.3111<br>(-1.9837)	16.8049<br>(2.2356)	34.8574<br>(7.4656)	0.6315<br>(7.4644)	0.4210<br>(7.6062)	-81.5104<br>(-3.5195)	0.1674<br>(0.5417)	0.1586<br>(1.2090)	0.2296<br>(1.8034)	0.0093<br>(3.6516)	0.0323
$\frac{GDP_j}{A_j D_{i,j}}$	0.1540<br>(4.6494)	0.0182<br>(0.5580)	-0.0411<br>(-0.3026)	14.5826<br>(1.9664)	34.9804<br>(7.5542)	0.5352<br>(6.2956)	0.3260<br>(5.7030)	-34.5256<br>(-1.7052)	0.9322<br>(4.3198)	-0.2876<br>(-4.3994)	-0.2097<br>(-3.3446)	0.0074<br>(3.2527)	0.0314
$B_{i,j}$	0.0570<br>(5.3576)	0.0339<br>(1.0439)	0.1188<br>(1.9000)	20.7151<br>(2.7070)	38.8034<br>(8.0946)	0.5866<br>(6.8340)	0.3500<br>(6.1780)	-25.2057<br>(-3.7354)	0.2072<br>(2.2947)	0.0033<br>(2.6990)	-0.0673<br>(-1.3607)	0.0298<br>(0.6144)	0.0320
$B_{i,j}p_j$	0.0400<br>(1.0093)	0.0360<br>(1.1092)	0.1402<br>(1.6833)	22.5310<br>(2.9529)	38.1605<br>(8.0844)	0.6224<br>(7.3597)	0.3391<br>(5.9250)	-34.3594<br>(-4.4866)	0.1590<br>(1.5393)	0.0039<br>(3.0682)	-0.0521<br>(-1.0860)	0.0783<br>(1.7655)	0.0320
$B_{i,j}GDP_j$	0.0390<br>(1.0030)	0.0357<br>(1.0985)	0.1435<br>(1.7325)	22.5302<br>(2.9541)	38.1463<br>(8.0873)	0.6228<br>(7.3648)	0.3391<br>(5.9284)	-34.5347<br>(-4.5214)	0.1564<br>(1.5209)	0.0040<br>(3.0765)	-0.0517<br>(-1.0749)	0.0793<br>(1.7735)	0.0320

Table S1: Exploring alternative spatial models. Results of econometric spatial model parameters upon calibration with different formulations of the spatial weight matrix W. The t-statistic signifying the difference of the estimated parameter from zero is denoted in the parentheses.

State	SSE	MSE	State	SSE	MSE
Alabama	0.4510	0.0226	Nebraska	1.9089	0.0954
Arizona	0.4385	0.0219	Nevada	0.9087	0.0454
Arkansas	0.6647	0.0332	New Hampshire	1.1838	0.0592
California	0.2967	0.0148	New Jersey	0.3612	0.0181
Colorado	0.4115	0.0206	New Mexico	1.1929	0.0592
Connecticut	0.4715	0.0236	New York	0.2002	0.0100
Delaware	1.6216	0.0811	North Carolina	0.3470	0.0173
Florida	0.2151	0.0108	North Dakota	2.0258	0.1013
Georgia	0.4114	0.0206	Ohio	0.1652	0.0083
Idaho	0.6780	0.0339	Oklahoma	0.4149	0.0207
Illinois	0.2012	0.0101	Oregon	0.4445	0.0222
Indiana	0.4450	0.0222	Pennsylvania	0.1703	0.0085
Iowa	0.5960	0.0298	Rhode Island	0.5536	0.0277
Kansas	0.7251	0.0363	South Carolina	0.4309	0.0215
Kentucky	0.4833	0.0242	South Dakota	2.7643	0.1382
Louisiana	0.4806	0.0240	Tennessee	0.5156	0.0258
Maine	0.5292	0.0265	Texas	0.3101	0.0155
Maryland	0.4596	0.0230	Utah	0.2846	0.0142
Massachusetts	0.4472	0.0224	Vermont	2.5937	0.1297
Michigan	0.6966	0.0348	Virginia	0.3948	0.0197
Minnesota	0.7136	0.0357	Washington	0.2351	0.0118
Mississippi	0.4534	0.0227	West Virginia	0.7062	0.0353
Missouri	0.3962	0.0198	Wisconsin	0.2886	0.0144
Montana	0.6400	0.0320	Wyoming	2.5167	0.1258

Table S2: Evaluation of the model's output. The model's output is compared against firearm ownership measured by the Gallup Poll Social Series Crime surveys through sum of squared errors (SSE) and mean of squared errors (MSE).

State	Firearm ownership	Background checks	Background checks per capita	Fraction of suicides with firearms
Alabama	0.2842	**0.0132**	**0.0131**	0.2661
Arizona	0.1989	0.2346	0.1929	0.3076
Arkansas	0.3228	**0.0405**	**0.0379**	0.2063
California	0.1140	0.0508	0.0521	0.3460
Colorado	0.1821	0.3032	0.2322	0.1894
Connecticut	**0.0154**	0.1583	0.1581	**0.0024**
Delaware	**0.0621**	0.2242	0.1989	**0.0010**
Florida	0.1886	0.4059	0.3666	0.3398
Georgia	0.2539	**0.0167**	**0.0142**	0.3139
Idaho	0.3364	0.1189	0.0920	0.0723
Illinois	0.0750	0.0638	0.0643	0.1650
Indiana	0.1981	0.2199	0.2090	0.1683
Iowa	0.1389	**0.0114**	**0.0108**	**0.0400**
Kansas	0.3526	0.1385	0.1330	0.1069
Kentucky	0.3394	**0.0426**	**0.0410**	0.1875
Louisiana	0.3889	**0.0352**	**0.0341**	0.2486
Main	0.1857	0.0977	0.0938	**0.0127**
Maryland	**0.0235**	**0.0076**	**0.0077**	0.0589
Massachusetts	**0.0010**	0.3499	0.3427	**0.0025**
Michigan	0.1467	0.1158	0.1158	0.2737
Minnesota	0.0975	0.0861	0.0776	0.1091
Mississippi	0.3401	**0.0127**	**0.0125**	0.2119
Missouri	0.2982	0.2300	0.2198	0.2241
Montana	0.3484	0.0999	0.0865	**0.0433**
Nebraska	0.1184	**0.0152**	**0.0140**	**0.0082**
Nevada	0.2566	0.1720	0.1433	0.1456
New Hampshire	0.0981	0.3308	0.3207	**0.0021**
New Jersey	**0.0024**	0.5076	0.5025	**0.0330**
New Mexico	0.3579	0.2428	0.2239	0.0768
New York	**0.0200**	0.3205	0.3182	0.1422
North Carolina	0.1583	**0.0123**	**0.0098**	0.2797
North Dakota	0.2261	0.1277	0.1077	**0.0017**
Ohio	0.1909	0.1698	0.1679	0.3213
Oklahoma	0.4330	0.1313	0.1227	0.2298
Oregon	0.3625	0.2504	0.2147	0.2198
Pennsylvania	0.1523	0.2783	0.2747	0.3018
Rhode Island	**0.0017**	0.3368	0.3355	**0.0010**
South Carolina	0.3369	0.0502	**0.0414**	0.1802
South Dakota	0.3011	0.1425	0.1234	**0.0010**
Tennessee	0.3110	0.1167	0.1036	0.3289
Texas	0.2335	0.0929	0.0749	0.4433
Utah	0.2510	**0.0215**	**0.0163**	0.0683
Vermont	0.0709	0.2077	0.2053	**0.0010**
Virginia	0.1664	0.2506	0.2290	0.2585
Washington	0.1646	0.3464	0.3116	0.2496
West Virginia	0.3771	0.0915	0.0935	0.1375
Wisconsin	0.1773	0.1831	0.1739	0.1591
Wyoming	0.2410	0.1043	0.0962	**0.0061**

Table S3: Stationarity of State-level time series.Reported p-values for the augmented Dickey-Fuller test, applied on raw data between January 2000 and December 2017. Values in bold represent a stationarity at a significant level of 0.05.

Date	Event	Date	Event
12/26/00	Edgewater Technology shooting, MA	4/21/13	Pinewood Village Apartments shooting, WA ∗
2/5/01	Navistar International shooting, IL	6/7/13	Santa Monica College shooting, CA ∗
7/8/03	Lockheed Martin shooting, MS	7/26/13	Todel Apartments shooting, FL
12/8/04	Damageplan show shooting, OH	9/16/2013	The Washington Navy Yard shooting, DC
3/12/05	Living Church of God shooting, WI	2/20/14	Cedarville Rancheria Tribal Office shooting, CA ∗
3/21/05	Red Lake Indian reservation shooting, MN	4/3/14	Fort Hood shooting II, TX ∗
1/30/06	Postal facility shooting, CA ∗	5/23/14	Santa Barbara County shooting, CA ∗
3/25/06	Capitol Hill shooting, WA ∗	10/24/14	Marysville-Pilchuck High School shooting, WA ∗
10/2/06	Amish School shooting, PA	6/11/15	Trestle Trail bridge shooting, WI
2/12/07	Trolley Square shooting, UT	6/17/15	Charleston Church Shooting, SC
4/16/07	Virginia Tech shooting, VA	7/16/15	Chattanooga military recruitment center, TN
10/7/07	Crandon duplex shooting, WI	10/1/15	Umpqua Community College shooting, OR ∗
12/5/07	Westroads Mall shooting, NE	10/31/15	Colorado Springs shooting rampage, CO
2/7/08	City council shooting, MO	11/27/15	Planned Parenthood clinic, CO
2/14/08	Northern Illinois University shooting, IL	12/2/15	Inland Regional Center shooting, CA ∗
6/25/08	Atlantis Plastics shooting, KY	2/20/16	Cracker Barrel shooting, MI
3/29/09	Carthage nursing home shooting, NC	2/25/16	Excel Industries mass shooting, KS
4/3/09	Immigration services center shooting, NY	6/12/16	Pulse nightclub shooting, FL
11/5/09	Army processing center shooting, TX ∗	7/7/16	Police protest march shooting, TX ∗
11/29/09	Pierce County coffee shop shooting, WA ∗	7/17/16	Baton Rouge police shooting, LO
8/3/10	Hartford Beer Distributors shooting, CT	9/23/16	Cascades Mall Macy's shooting, WA ∗
1/8/11	Safeway parking lot shooting, AZ ∗	1/6/17	Fort Lauderdale airport shooting, FL
9/6/11	IHOP shooting, NV ∗	4/18/17	Fresno downtown shooting, CA ∗
10/12/11	Salon Meritage shooting, CA ∗	5/12/17	Rural Ohio nursing home shooting, OH
2/21/12	Su Jung Health Sauna shooting, GA	6/5/17	Florida awning manufacturer shooting, FL
4/2/12	Oikos University shooting, CA ∗	6/7/17	Pennsylvania supermarket shooting, PA
5/30/12	Café Racer shooting, WA ∗	6/14/17	San Francisco UPS shooting, CA ∗
7/20/12	Century 16 movie theater shooting, CO	10/1/17	Las Vegas Strip massacre, NV ∗
8/5/12	Sikh temple of Wisconsin shooting, WI	10/18/17	Edgewood business park shooting, MD
9/27/12	Accent Signage Systems shooting, MN	11/1/17	Walmart shooting in suburban Denver, CO
12/14/12	Sandy Hook Elementary School shooting, CT	11/5/17	First Baptist Church shooting, TX ∗
3/13/13	Mohawk Valley shootings, NY	11/14/17	Rancho Tehama shooting spree, CA ∗

Table S4: Mass shootings in the U.S. between January 2000 and December 2017. The data were was obtained from Mother Jones and exclude the U.S. Territories. ∗ denotes events that took place in the West and Southwest (24 out of 64 events in total).

	Firearm ownership	Mass shootings	Media output
Firearm ownership	–	0.0464 ∗ (0.0128)	0.0579 ∗ (0.0026)
Mass shootings	0.0098 (0.5993)	–	0.0137 (0.4665)
Media output	0.0302 ∘ (0.0755)	0.0231 (0.1831)	–

Table S5: Causal analysis in the absence of τ and η. Transfer entropy was computed between firearm ownership, mass shootings, and media output, where the time series for firearm ownership was estimated from model neglecting τ and η. Rows are sources and columns are targets. The numbers in parentheses denote the p-value obtained from a permutation test.

.

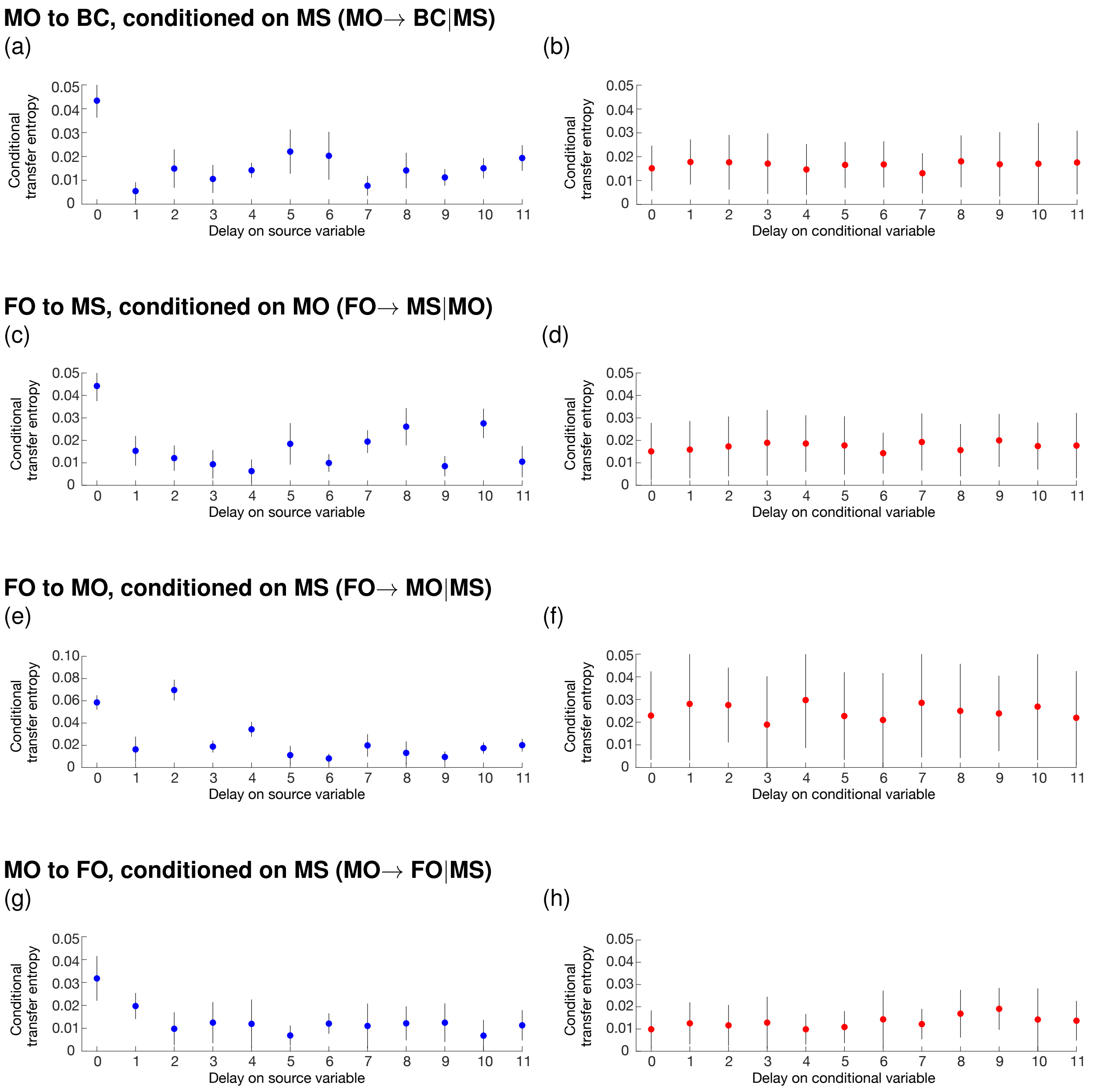

Figure S5: Delay analysis for causal links. Conditional transfer entropy was computed for national level time series with delays on the source variable time series (a,c,e,g) or the conditional variable (b,d,f,h). The round markers represent the mean of the delay, computed over the 12 delays of the source variable or conditional variable. The vertical bars represent $\pm$ one standard deviation.

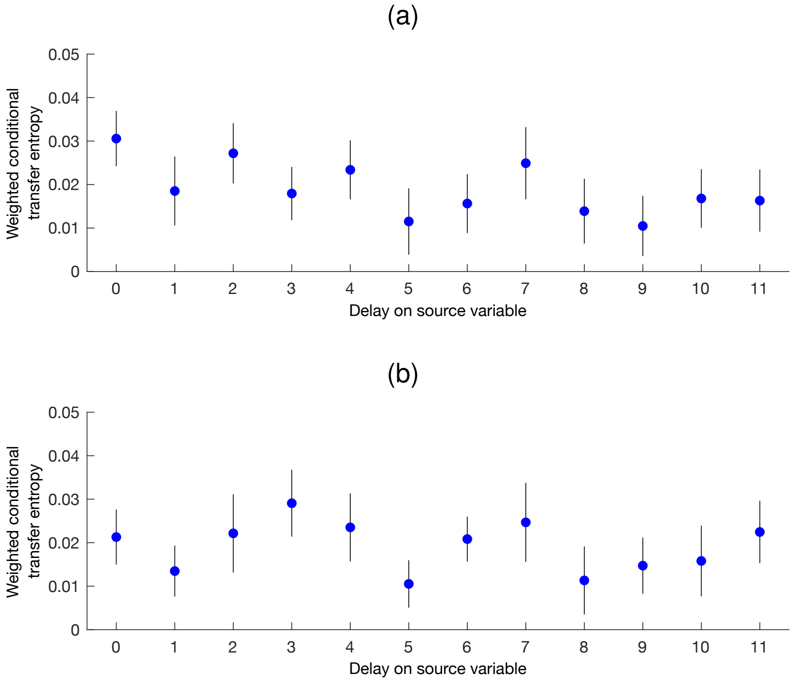

Figure S6: Delay analysis in permissive and restrictive states. Delayed conditional transfer entropy from FO to MO was computed separately for permissive states (a) and restrictive states (b). The round markers represent the mean value of transfer entropy across states, weighted by their population size. The vertical bars represent $\pm$ one standard deviation.

	Firearm ownership	Mass shootings	Media output
Firearm ownership	–	0.0123 (0.4819)	0.0116 (0.5284)
Mass shootings	0.0144 (0.4056)	–	0.0057 (0.8157)
Media output	0.0240 (0.1540)	0.0123 (0.4877)	–

Table S6: Causal analysis in the absence of spatial associations. Transfer entropy was computed between firearm ownership, mass shootings, and media output, where the time series for firearm ownership was generated from model neglecting spatial interactions ($W = 0$). Rows are sources and columns are targets. The numbers in parentheses denote the p-value obtained from a permutation test.

.
